# Functional Annotation and Curation of Hypothetical Proteins Present in A Newly Emerged Serotype 1c of *Shigella flexneri*: Emphasis on Selecting Targets for Virulence and Vaccine Design Studies

**DOI:** 10.3390/genes11030340

**Published:** 2020-03-23

**Authors:** Tanuka Sen, Naresh K. Verma

**Affiliations:** Division of Biomedical Science and Biochemistry, Research School of Biology, The Australian National University, Canberra, ACT 2601, Australia; tanuka.sen@anu.edu.au

**Keywords:** hypothetical proteins, *Shigella flexneri*, functional elucidation, protein function, virulence, vaccine development

## Abstract

*Shigella flexneri* is the principal cause of bacillary dysentery, contributing significantly to the global burden of diarrheal disease. The appearance and increase in the multi-drug resistance among *Shigella* strains, necessitates further genetic studies and development of improved/new drugs against the pathogen. The presence of an abundance of hypothetical proteins in the genome and how little is known about them, make them interesting genetic targets. The present study aims to carry out characterization of the hypothetical proteins present in the genome of a newly emerged serotype of *S. flexneri* (strain Y394), toward their novel regulatory functions using various bioinformatics databases/tools. Analysis of the genome sequence rendered 4170 proteins, out of which 721 proteins were annotated as hypothetical proteins (HPs) with no known function. The amino acid sequences of these HPs were evaluated using a combination of latest bioinformatics tools based on homology search against functionally identified proteins. Functional domains were considered as the basis to infer the biological functions of HPs in this case and the annotation helped in assigning various classes to the proteins such as signal transducers, lipoproteins, enzymes, membrane proteins, transporters, virulence, and binding proteins. This study contributes to a better understanding of growth, survival, and disease mechanism at molecular level and provides potential new targets for designing drugs against *Shigella* infection.

## 1. Introduction

*Shigella* spp are causative agent of an extreme enteric infection known as shigellosis; they are Gram-negative facultative anaerobes that belong to the family of *Enterobacteriaceae* and are closely related to *Escherichia coli* [1]. In 2015, *Shigella* was identified as the second most prominent cause for diarrheal deaths on a global scale [2]. Spreading of the infection is generally limited to the intestinal lining, where it leads to colonic inflammation, mucosal ulceration, and a loss in intestinal barrier function. *Shigella* is transmitted through the fecal-oral route or through ingestion of contaminated food and water [3]. In most cases, *Shigella* spp. causes a self-limiting disease that can be effectively treated by oral rehydration or antibiotics, though it can be fatal in the very young and in infected individuals who are immunocompromised or do not have access to adequate medical treatment [4,5]. There is a steady rise in the number of shigellosis cases caused by antibiotic-resistant *Shigella* strains, which has become a growing concern. 

Clinical symptoms of shigellosis range from mild watery diarrhea to a bloody mucoid diarrhea along with painful abdominal cramps and fever. The range of clinical symptoms is related to both the immune status of the host and the causative *Shigella* species, which differ in the presence of some critical virulence factors, including Shiga toxin [5,6,7]. A major complication in infants and children is toxic megalocolon, while after clearance of the infection, other possible complications include hemolytic-uremic syndrome, characterized by renal failure, low platelet and red blood cell levels, and a 35% fatality rate, as well as post reactive arthritis, where patients may suffer from chronic arthritis of the joints for years after a shigellosis episode [7,8]. *Shigella* has a very low infectious dose, estimated to be 10 to 100 bacteria, and it remains a major public health concern with an estimated 165 million cases occurring worldwide every year, including up to 100,000 deaths, particularly in children under 5 years of age [9,10].

The genus *Shigella* has been divided into four species namely: *S. flexneri, S. sonnei, S. dysenteriae, and S. boydii*. They have been further categorized into serotypes based on the biochemical differences and variations in their O-antigen [11]. So far various research groups have reported 19 different serotypes of *Shigella flexneri* [12]. Since the 1990s several new serotypes have been reported and added into the list of 19 known serotypes, which include 7b, 1d, Xv, 4s, 4av, and 1c strains [13,14]. 

The first discovery of the newly emerging 1c strain was in 1989 in Bangladesh. Its basic tetrasaccharide repeating unit contains a disaccharide linked to the N-acetylglucosamine, whereas serotypes 1a and 1b strains contain only a single glucosyl group at the same position [14]. Its name was coined by Wehler and Carlin in 1988 based on its similarity to other serotype 1 strains [14].

Since then serotype 1c has been isolated and reported in other countries, mainly Egypt, Indonesia, Pakistan, and Vietnam [15,16,17]. Serotype 1c has been shown to be the most prevalent *S. flexneri* serotype in Bangladesh and Northern Province of Vietnam [16,18]. As reported by Talukder et al. in 2003 the prevalence of this strain in Bangladesh alone increased from 0 to 56% by 2001 [18]. The Sereny test conducted in this study also revealed that 88% of the serotype 1c strains were invasive [18]. 

Shigellosis in the developing countries especially Asia is primarily caused by *S. flexneri* and is responsible for approximately 10% of all diarrheal episodes among children of <5 years [19]. A vaccine for *Shigella* has not yet been licensed, partly because of the large repertoire of its serotypes that need to be targeted in order for the vaccine to be globally effective. Because of the rise in the cost of treatment, increased antibiotic resistance and the tenacity of poor hygiene and sanitation problems, the efficacy of existing antimicrobial treatments has been compromised [20]. 

Numerous genes present on chromosome and the virulence plasmid have been identified to be involved in the pathogenesis, growth, and survival of *Shigella* [21,22,23,24,25,26,27]. Nonetheless, many loci with plausible protein coding genes are inadequately understood for their presence and consequent relationships in the life cycle of *Shigella*, thus being annotated as “Hypothetical proteins” [28]. A hypothetical protein is one that is predicted to be encoded by a known open reading frame, but its putative function is not known as there are no experimental evidences [28]. Approximately half of the protein encoding genes in most genomes are classified as hypothetical proteins (HPs) and this category of proteins probably have their own significance in the total proteomic platform of an organism [29]. Accurate annotation of HPs present in a pathogen leads to a better understanding of the virulence mechanisms, discovery of new structures, additional protein pathways and functions [29]. HPs may perhaps play essential roles in the growth, survival, and the disease advancement. Additionally, they may also function as genetic markers and pharmacological targets for generating new drugs and treatments against the pathogen [30,31]. To categorize a HP as an essential gene and as a novel drug target, it should first be pathogen specific i.e., non-homologous to the host and secondly it should be a vital gene involved in the replication, survival, virulence, or growth of the pathogen [32].

Bioinformatics in the recent years has enhanced our understanding of the structure function relationships of proteins. The benefit of these being more cost effective and less time consuming compared to the traditional in vitro methods. Functional characterization of HPs using various structure and sequence-based bioinformatic tools can help in classifying these proteins into diverse functional classes, which can give more insight into their activities, structures, and their roles in the metabolism [33]. Elucidation of roles of these HPs from several pathogenic organisms such as *Vibrio cholerae O139, Chlamydia trachomatis, Neisseria meningitidis, Mycobacterium tuberculosis, Haemophilus influenzae, Helicobacter pylori,* and others using bioinformatic tools have already been reported [34,35,36,37,38,39].

In this study, several enhanced and up-to-date bioinformatics tools were employed to allocate functions of a number of HPs from the genome of *S. flexneri* 1c strain Y394 [12]. It consists of 4,584,634 bp in a single chromosome containing 4958 genes encoding 4170 proteins [12]. Among these, the functions of 721 proteins have not been functionally characterized and are termed as HPs. The amino acid sequences of these HPs were evaluated using a combination of latest bioinformatics tools based on homology search against functionally identified proteins, domain analysis, physiochemical factors, subcellular localization, and prediction of virulence. Among the 721 HPs, putative functions of a total of 246 HPs have been assigned in this study. The annotation helped in assigning various classes to the proteins such as signal transducers, lipoproteins, enzymes, membrane proteins, transporters, virulence, and binding proteins. We believe that this analysis will expand our knowledge regarding the functional roles of HPs present in *Shigella* and provide an opportunity to unveil a number of potential targets. These identified targets can then be validated with further experiments, which will eventually help in developing novel drugs or vaccines to treat or prevent shigellosis [40].

## 2. Materials and Methods 

**Figure 1:**
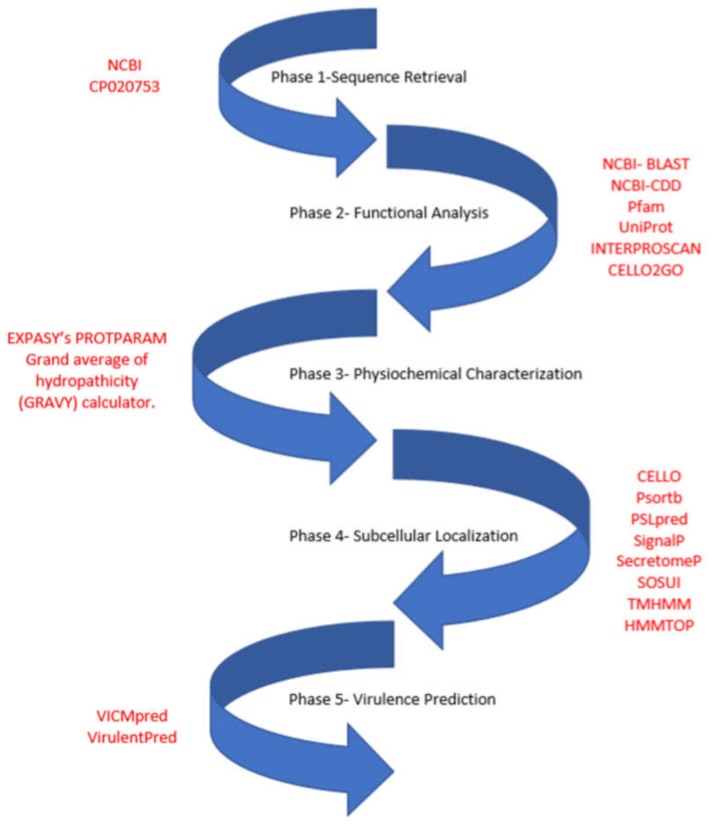
Workflow used for the functional annotation of hypothetical proteins in the *Shigella flexneri* 1c genome. The entire workflow can be divided into five phases, involving sequence retrieval of the HPs from the genome; functional analysis by identifying conserved domains and GO annotations; followed by analyzing their physiochemical characterization, subcellular localization, and transmembrane helices; and lastly determining if they are involved in virulence of *S. flexneri*.

The *S. flexneri* 1c genome with accession number- CP020753 at GenBank served as data source. The sequences of 721 hypothetical proteins were extracted from here for further functional interpretations using in silico methods. The entire work scheme illustrating all bioinformatics tools used is shown in Figure 1. The entire workflow can be divided into five phases, involving sequence retrieval of the HPs from the genome; functional analysis by identifying conserved domains and their Gene ontology (GO) annotation; followed by analyzing their physiochemical characterization, subcellular localization and transmembrane helices; and lastly determining if they are involved in virulence of *S. flexneri*.

### 2.1. Functional Assignment and Domain Analysis

Assigning functions to all the 721 HPs of Y394 was carried out using various publicly accessible bioinformatic tools and databases namely NCBI- Protein BLAST, Pfam, Conserved domain database, and InterProScan. Domains are structural and/or functional units of proteins, that are conserved in each protein family/superfamily [40]. A higher amount of conservancy is found in domains/folds compared to the entire sequence [41]. The identification of domains that occur within proteins can therefore provide insights into their function [41].

NCBI’s Protein BLAST and CDD database were used to predict homologous proteins with same or similar functions and presence of conserved domains [42,43]. Pfam is a large collection of protein families (annotated), each characterized by hidden Markov models and multiple sequence alignments, with this the proteins sequences of HPs can be analyzed [44]. InterProScan helped in identifying motifs and domains by combining various protein signature recognition methods [45]. CELLO2GO tool was used to identify the GO annotation and subcellular localization of a particular HP. It uses BLAST to analyze the target protein to homologous sequences that are already GO annotated, and classifies the query sequence to their GO categories, i.e., molecular function, biological process and cellular component. The results are summed and presented as pie charts representing possible functional annotations for the queried protein [46]. All these tools helped in categorizing HPs into functional classes. 

### 2.2. Physiochemical Characterization

Analysis of the physiochemical parameters was carried out using Expasy’s ProtParam tool [47], factors like molecular weight, number of amino acids, isoelectric point, extinction coefficient, and the grand average of hydropathicity (GRAVY) were examined.

### 2.3. Subcellular Localization Analysis

For determining the subcellular localization of the HPs, three different bioinformatic tools were used, namely CELLO, PSORTb, and PSLpred, these tools are based on support vector machine (SVM) prediction system for predicting the location of proteins [48,49,50]. Out of the three, PSLpred is believed to have an accuracy of 91% and consists of a hybrid-SVM-based prediction method [50]. SOSUI was used to distinguish between soluble and membrane proteins, it also predicts transmembrane helices of the membrane proteins [51]. 

Definite prediction of transmembrane helices and topology of the membrane bound HPs were done using two bioinformatic tools namely TMHMM and HMMTOP, which utilizes hidden Markov model to predict the presence of transmembrane helices [52,53]. Presence of signal peptide in HPs was predicted using SingnalP 5.0 online tool [54] and SecretomeP 2.0 was used to analyze if the HPs were involved in non-classical secretory pathway [55].

### 2.4. Virulence Factor Prediction

Two bioinformatic tools were used to predict if the HPs present can be categorized as virulence factors. VICMpred and VirulentPred that are based on SVM method were used; these use dipeptide composition, amino acid composition, and other patterns to predict virulence factors, possessing an accuracy of 70–80% [56,57]. VICMpred classifies proteins into categories like information molecule, cellular process, virulence factor and metabolism molecule; whereas VirulentPred can only distinguish proteins in two classes namely virulent and non-virulent. 

## 3. Results and Discussion

### 3.1. Sequence Analysis and Functional Annotation

**Figure 2:**
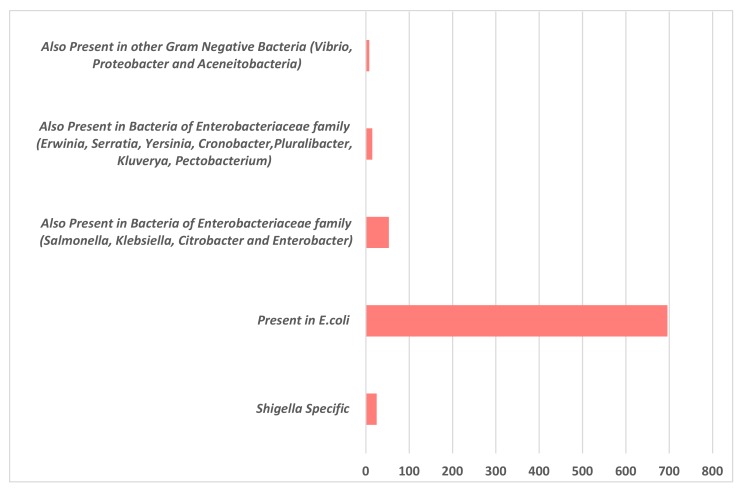
NCBI Protein BLAST of the 721 hypothetical proteins (HPs) present in *Shigella flexneri* 1c strain showed that these HPs were also present in other Gram-negative bacteria, majority of them being present in different pathogenic and non-pathogenic *E. coli* strains, followed by other Gram-negative pathogenic bacteria belonging to the family of *Enterobacteriaceae.*

There has been no experimental analysis to characterize the hypothetical proteins present in *S. flexneri* 1c strain, which has previously been sequenced, hence an effort was made to annotate the function of these HPs, using an in silico approach. Sequences of all the 721 HPs were analyzed for the presence of functional domains using four bioinformatics tools namely CDD-BLAST, Pfam, InterProScan, and SCANPROSITE. During the analysis, it was found that most of these HPs were also present in other Gram-negative bacteria, especially *E. coli,* which is the closest relative of *Shigella*. Most of the HPs were found in various members of the *Enterobacteriaceae* family. There were about 25 HPs that were found to be specific to *Shigella* spp, out of which one of the HP was only specific to *S. flexneri* 1c strain. Detailed representation of these HPs being present in other Gram-negative organisms is shown in Figure 2. 

**Figure 3:**
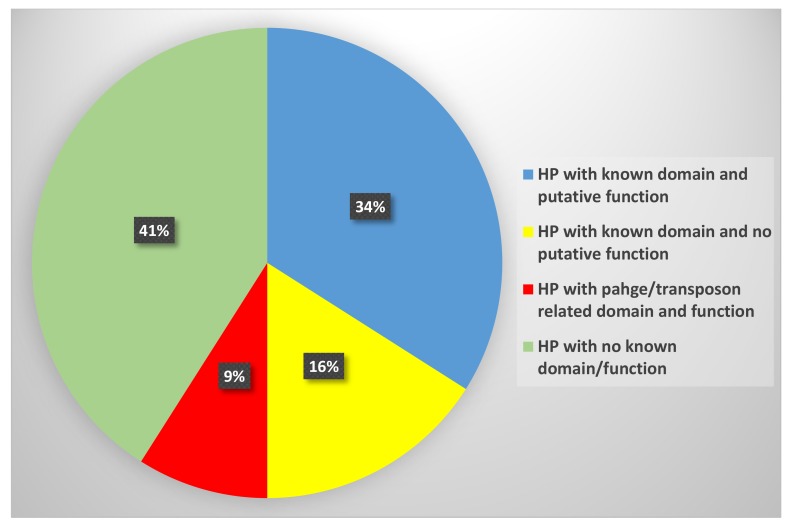
The distribution of 721 HPs present in the *Shigella flexneri* 1c genome. Sequence analysis of the 721 HPs was carried out using four bioinformatics tools, namely CDD-BLAST, Pfam, InterProScan, and SCANPROSITE. This resulted in 293 HPs (41%) with no specific conserved domains and the remaining 428 HPs, for which specific domains were assigned. These consisted of 246 HPs with both known domain and putative function, 119 HPs with only known domains, and 62 HPs with domains related to bacteriophage genes like tail/head/assembly proteins or the transposon genes.

Out of these 721 HPs, for about 293 proteins, there were no specific conserved domains found, though BLAST did give a few similarity results with homologous proteins. In the remaining 428 HPs, specific domains were assigned, majorly consisting of 246 HPs to which both domains and putative functions could be assigned. About 119 HPs have domains, mostly DUF (domain of unknown function), but their functions are not yet known or not characterized. Lastly, there were 62 HPs with domains that were related to bacteriophage genes like tail/head/assembly proteins and the transposon genes (Figure 3).

**Figure 4:**
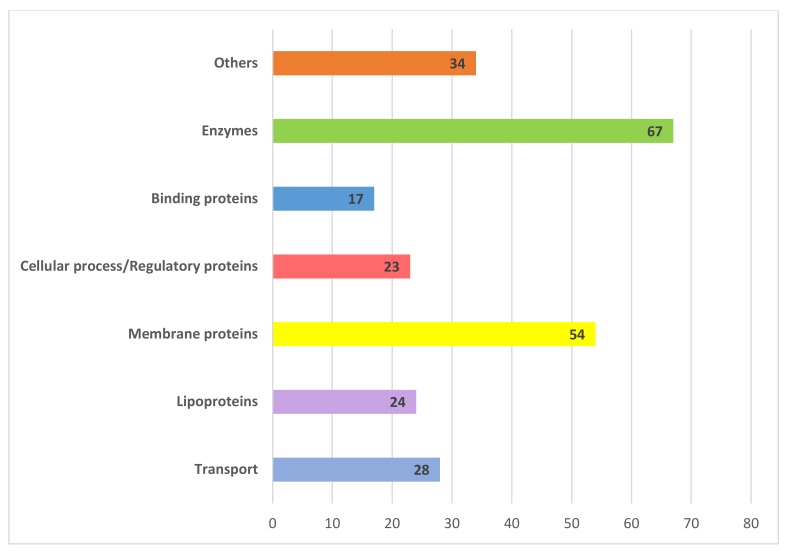
The distribution of 246 hypothetical proteins into different functional groups based on their predicted protein domains and families. Sequence analysis carried out using tools like CDD-BLAST, Pfam, InterProScan, and SCANPROSITE, helped in categorizing the HPs into different functional classes. Majority of these functional classes were binding proteins, enzymes, transport proteins, lipoproteins, membrane proteins, and proteins involved in various cellular/regulatory processes.

**Figure 5:**
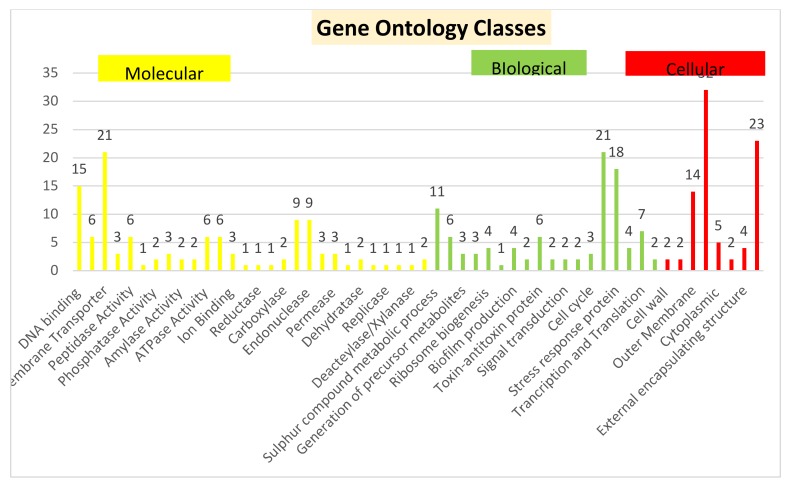
Gene ontology of 246 hypothetical proteins in *S. flexneri* strain 1c. The proteins were classified according to biological, cellular and molecular functions based on the domain analysis done with Cello2Go, UniProt and InterProScan (Some functions fall in more than one gene ontology class).

The 246 HPs with known domains and putative function were found to be present in various functional categories namely binding proteins, enzymes, transport proteins, lipoproteins, membrane proteins, and proteins involved in various cellular/regulatory processes. Description of the major functional groups of these 246 HPs has been discussed in detail and is illustrated in Figure 4. Hence, only these 246 proteins with known domains and some putative function were considered for further bioinformatics analysis. Domain analysis results of these HPs are listed in Table S1. Gene ontology analysis of these proteins was based on their functional domain identification and also a bioinformatic tool Cello2Go was used for confirming the gene ontology classes, result of this is illustrated in Figure 5. 

### 3.2. Transport Proteins

Proteins that are involved in transport are considered to play an essential role in bacterial metabolism, they take part in excretion of waste products, uptake of nutrients, exclusion of antibiotic drugs, and maintaining the cytoplasmic balance of protons and salts needed for the growth and development of the bacteria [58,59]. Most of these transport proteins have been identified to be involved in virulence and fundamental to intracellular survival of pathogens [60]. We successfully identified about 21 putative transporters, 4 signal transduction proteins, and 3 carrier proteins among the HPs (Table S1).

The protein ATH68112.1 was predicted to be a member of the *EamA* family - Drug/metabolite transporter (DMT) superfamily, these are assumed to be involved in the export of metabolite and drugs in prokaryotes [61]. Protein ATH67957.1 was predicted to be an autoinducer 2 ABC transporter substrate binding protein. Autoinducers act as signaling molecules that help bacteria in communicating with one another through quorum sensing [62]. Proteins like ATH67303.1, ATH70219.1, and ATH70237.1 (Table S1) were predicted to be transporters involved in signal transduction, these proteins are believed to help the bacteria sense their environmental parameters like temperature, pH, light, etc., [63]. Proteins involved in signaling have emerged as attractive antibacterial drug targets, as impairing these can affect both upstream and downstream physiological functions of the bacteria [64]. Hypothetical proteins like ATH67468.1, ATH67810.1, ATH68182.1, and ATH68515.1, were predicted to be transporters/carriers of specific molecules namely amino acids, manganese, copper, and Sulphur, respectively (Table S1). Around four of the proteins were predicted to homoserine/threonine transporters namely ATH68713.1, ATH69323.1, ATH70469.1, and ATH70687.1 (Table S1).

### 3.3. Binding Proteins 

Seventeen HPs were annotated as binding proteins in which four were RNA binding, seven DNA binding, three heavy metal binding, one peptidoglycan binding, and two ligand/substrate binding proteins.

HPs ATH66955.1, ATH68077.1, ATH68551.1, ATH68741.1, ATH68742.1, ATH69585.1, and ATH70244.1 were predicted as DNA binding proteins (Table S1). DNA binding proteins bind specifically to double or single stranded DNA and regulate expression of genes and nucleases [65]. DNA binding proteins also play a role in virulence, the best known example of it being the HU protein that binds to various genes and controls motility, growth, metabolism, and virulence in *Vibrio parahaemolyticus* [66]. Proteins ATH68737.1, ATH69313.1, ATH69782.1, ATH68961.1 were predicted as RNA-binding proteins (Table S1). It is assumed that RNA-binding proteins also contribute to the survival of the organism and play a role in controlling the virulence factors [67].

We discovered a tetratricopeptide repeat (TPR) present in tree binding proteins ATH66845.1, ATH68837.1, and ATH69836.1 (Table S1). TPR is a structural motif that is involved in the assembly of multiprotein complexes, protein–protein interactions. TPR-containing proteins play vital roles in various cellular process and are believed to play a significant role in virulence [68]. The HP ATH69109.1 was predicted to be a peptidoglycan-binding domain-containing protein *LysM,* it is also known as the lysin motif, binding to peptidoglycan and chitin, having multiple functions in bacteria, animals, and plants [69]. This domain is present in many proteins that act as virulence factors of various human bacterial pathogens; *Staphylococcus aureus* produces five *LysM* proteins which are all involved in virulence [70]. 

### 3.4. Lipoproteins 

Lipoproteins are the peripheral membrane proteins that are associated with the cell membrane by N-terminally linked fatty acids [71]. Bacterial lipoproteins have been shown to be involved in signal transduction, conjugation, sporulation, nutrient uptake, transport, help in folding of proteins, and also take part in development of antibiotic resistance [72]. In pathogens, lipoproteins play vital roles in virulence associated functions namely by aiding in adhesion to host, modulating inflammatory processes and in transferring virulence factors into the host [73]. We found 24 lipoproteins from the group of 246 HPs predicted in this study, these can be considered as potential targets for further experimental analysis, as lipoproteins are such crucial for the pathogen. Analysis of antigenic membrane proteins led to the identification of a number of surface exposed lipoproteins, that are immunogenic and can be used as potential vaccine candidates [74]. In our analysis, we found a HP ATH66743.1, that is predicted to be a putative surface-exposed outer membrane lipoprotein, *YaiW* belonging to DUF1615 protein family. 

### 3.5. Membrane Proteins

We found about 54 HPs that were predicted as membrane proteins in this analysis, these included general membrane proteins, integral inner membrane proteins, and outer membrane proteins (Table S1). Gram-negative bacteria are surrounded by both an outer membrane and an inner membrane. Membrane proteins aid bacterial cells in numerous ways, they are involved in solute and protein translocation, assembly of membrane, formations of wall and capsules, signal transduction, metabolite transport, also have receptors for bacteriophage, colicins, and antibiotics [75,76]. Most of the bacterial surface membrane proteins are believed to play a role in pathogenicity, are immunogenic, and act as excellent targets for vaccine development [77]. 

### 3.6. Enzymes 

Bacterial enzymes aid in survival of the pathogen in their host because they provide essential growth factors, nutrients, and also are involved in the pathogenesis [78]. They play a role in host–pathogen interaction and alter the host environment to suit the pathogen growth and virulence [78]. We characterized 67 enzymes in the group of 246 HPs in our analysis, majority of them falling into recognized enzyme classes namely oxidoreductases, transferases, hydrolases, isomerases, and ligases (Table S1). 

Hydrolases are enzymes that catalyze the hydrolysis of a chemical bond, mostly involved in cleavage of different peptidoglycan bonds in bacteria [79]. Hydrolases are associated with various virulence factors and are predicted to play a role in invasion and evasion of the host defense system [79]. In the present study, we identified seven hydrolase enzymes (ATH67540.1, ATH68089.1, ATH68145.1, ATH68250.1, ATH68320.1, ATH68497.1, and ATH68856.1), namely belonging to subfamilies like alpha/beta hydrolases, dNTP triphosphohydrolase, HAD-IIB family murein hydrolase and glycoside hydrolase. Similarly, we identified ten transferase enzymes, these enzymes are important for bacterial pathogens, as they are involved in spore germination, synthesis of lipoproteins and virulence [80]. Protein ATH67546.1 and ATH68125.1 were predicted to be an acetyltransferase, belonging to the Acyl_transf_3 family; these proteins transfer acetyl group to a substrate and are involved in reactions related to the development of antibiotic resistance [81]. HP ATH70596.1 was predicted to be a glycosyltransferase, these are assumed to be involved in lipopolysaccharide and extracellular polysaccharide biosynthesis [82]. Additional transferases identified were phosphotidytransferases (ATH67855.1, ATH68146.1, ATH68531.1), sulphurtransferase (ATH68374.1, ATH68662.1), thiosulphate sulphurtransferase (ATH68957.1), and phosphopantetheiyl transferase (ATH69906.1). 

Six different oxidoreductase enzymes were predicted in this group of HPs (ATH67165.1, ATH70538.1, ATH68061.1, ATH68075.1, ATH69025.1, and ATH69677.1). Oxidoreductases are known to be involved in bacterial pathogenicity as they form the disulphide bonds, which in turn maintain stability and rigidity of many extracellular proteins including virulence proteins [83]. We also identified two ligases and one isomerase enzyme. Proteins ATH67096.1 and ATH67099.1 were identified as putative tRNA ligases and protein ATH70056.1 was predicted to be a xylose isomerase belonging to *AP2Ec* family. Formation of a chemical bond by joining two large molecules is catalyzed by ligase enzyme whereas isomerases catalyze structural rearrangements within one molecule [84,85]. Few other important enzymes predicted in this group of HPs were kinases (ATH67373.1, ATH68297.1), permeases (ATH68327.1, ATH68713.1, ATH69323.1), amylases (ATH68947.1), endonucleases (ATH69573.1), primase/helicases (ATH67175.1), replicases (ATH70660.1), etc. 

### 3.7. Cellular Process/Regulatory Proteins 

A total of 23 HPs were predicted to be involved in various cellular and regulatory processes, which are essential for the growth and survival of the pathogen and therefore can be considered as potential targets for drug development (Table S1). Proteins involved in bacterial cellular processes, take part in growth, replication, and survival of the bacteria and the regulatory proteins help the pathogen to adapt to the host niche, they control the bacterial response to the environmental changes like stress and nutritional conditions [86]. 

In our analysis, we identified about eight proteins that are involved in various stress responses, namely pH stress (ATH66527.1, ATH67887.1, and ATH69301.1), osmotic stress (ATH69181.1), and DNA damage stress (ATH68190.1). Proteins ATH66865.1 and ATH66913.1 were predicted to be competence ComEA protein and NfeD family protein (nodulation efficiency protein), respectively. ComEA protein enhances the bacterial competence, which is the ability of a cell to take up exogenous DNA, this protein is also essential for DNA binding and transport [87]. NfeD family proteins on the other hand are extensively distributed throughout prokaryotes and are always linked with genes encoding stomatin-like proteins (slipins), though functions of these proteins remain largely unknown [88]. 

There are seven HPs that were predicted as transcriptional regulator proteins (ATH66868.1, ATH67828.1, ATH68741.1, ATH68742.1, ATH70611.1, ATH69450.1, and ATH69554.1), Table S1. These proteins are considered important in regulating transcription of particular genes, they do this by binding to the DNA and blocking/helping the transcription process [89]. ATH66868.1 being a HTH-type transcriptional regulator *SgrR*, activates the small RNA gene *SgrR*, under stress and non-stress conditions and controls its level of synthesis, thereby acting as a sensor of the intracellular buildup of phospho-glucose [90]. 

### 3.8. Physiochemical and Subcellular Localization Analysis

Peptides and proteins possess different physicochemical properties that exert critical impacts on their activity, structure, and thus biological function. These physiochemical parameters can be calculated and predicted to better understand a molecule’s function. ExPASy’s ProtParam tool was used to study physiochemical properties of the HPs which included number of amino acids, molecular weight, theoretical pI, extinction coefficient, and grand average of hydropathicity (GRAVY) [47]. Results of this analysis are listed in Table S2. Determining the sub-cellular localization of proteins is essential to decide if they can be used as vaccine or drug targets. Proteins that reside in the cytoplasm are considered as potential drug targets, while the surface membrane proteins can serve as potential vaccine candidates [48]. 

Subcellular localization of the HPs was determined using three different tools based on hidden Markov model namely PSORTb, CELLO, and PSLpred [48,49,50]. This analysis helped in grouping the proteins according to their location—cytoplasmic, periplasmic, extracellular, inner membrane, or outer membrane. HMMTOP, SOSUI, and TMHMM were used for predicting if the HPs proteins were soluble or membrane proteins and to determine the presence of transmembrane helices [50,51,52]. About 60 predicted membrane proteins had transmembrane helices ranging between 1–10 (Table S3).

SignalP 4.1 was used to predict the signal peptide and SecretomeP 2.0 was utilized for the identification of proteins involved in non-classical secretory pathway [54,55]. This analysis helped in identifying which HPs had a signal peptide attached and which ones were secretory in nature. Detailed results for each of these predictions are summarized in Table S3.

### 3.9. Virulence Factor Prediction

Each pathogen be it fungi, protozoa, viruses, or bacteria produces virulence factors that enable them to cause infection and impair the host. Virulent factors can be predicted using bioinformatic tools like VICMpred and VirulentPred which are based on PSI-Blast and support vector machine (SVM) method for prediction of virulent protein sequences [56,57]. Predictions like these can help in selecting interesting vaccine/drug targets. In this study, VICMpred and VirulentPred tools were employed to analyze the 246 HPs, out of which seven proteins (ATH66941.1, ATH67162.1, ATH68055.1, ATH68611.1, ATH70274.1, ATH70275.1, and ATH70596.1) were found to be virulent by both the software. Rest of the HPs were predicted to be involved in either metabolic or cellular processes. Detailed results of this prediction are listed in Table S4.

## 4. Conclusions

Extensive genome sequencing endeavors have generated large amounts of data at both proteomic and genomic level, although research on hypothetical proteins has been largely ignored. Characterization of HPs can pave the way for better understanding of bacterial metabolic pathways, disease progression, drug development, and disease control strategies. In this study, an in silico approach comprising a combination of various bioinformatics tools/databases was used for functional characterization of the HPs present in *S. flexneri* 1c strain Y394. Using this strategy, all 721 HPs from Y394 were primarily analyzed and then out of that, 246 HPs were taken forward for further analysis based on their domains and putative functions which included analyzing their physiochemical parameters, sub-cellular localization, and virulence prediction. This in silico study eventually helped in selecting and prioritizing targets for further experimental analysis, which included various assays to test the predicted function of HPs. Further research on HPs in the future can transform our understanding about the mechanism of disease, diagnosis, disease treatment, and vaccine design not only in *Shigella* but also in other medically significant bacterial pathogens. 

## Supplementary Materials

The following are available online at https://www.mdpi.com/2073-4425/11/3/340/s1. Table S1: Functional domain analysis of 246 HPs present *S. flexneri* 1c Y394, using four bioinformatics tools namely CDD-BLAST, Pfam, InterProScan, and SCANPROSITE. Table S2: Analysis of physiochemical characteristics for the 246 HPs present in *S. flexneri* 1c Y394. ExPASy’s ProtParam tool was used to study physiochemical properties of the HPs, which included number of amino acids, molecular weight, theoretical pI, extinction coefficient, and grand average of hydropathicity (GRAVY). Table S3: Analysis of the subcellular localization and presence of transmembrane helices present in 246 HPs. Bioinformatic tools PSORTb, CELLO, and PSLpred were used for subcellular localization; HMMTOP, SOSUI, and TMHMM were used for identifying soluble or membrane proteins with presence or absence of transmembrane helices; SignalP 4.1 and SecretomeP 2.0 were used for predicting signal peptide and secretory proteins. Table S4: Virulence factor prediction of the 246 HPs. Prediction and analysis were done using bioinformatic tools like VICMpred and VirulentPred which are based on PSI-Blast and support vector machine (SVM) method.
