# Supplementary material for: Functional Annotation and Curation of Hypothetical Proteins Present in A Newly Emerged Serotype 1c of Shigella flexneri: Emphasis on Selecting Targets for Virulence and Vaccine Design Studies
Source: Genes (Basel). 2020 Mar 23;11(3):340. doi: 10.3390/genes11030340 (PMC7141135; doi:10.3390/genes11030340)
Supplement: Supplementary file 1 [file genes-11-00340-s001.zip › Supplementary Table 1 .pdf]

Supplementary Table 1- Functional domain analysis of 247 HP's present *S. flexneri* 1c Y394, using four bioinformatics tools namely CDD-BLAST, Pfam, InterProScan, and SCANPROSITE.

S.No	Start-Stop/ Acc ID	Protein ID	BlastP result	Functional Domain- CDD-BLAST/Pfam/Uniprot/InterProScan
1.	11355-10642	ATH66527.1	msyB Acidic protein	DUFF 3944; Ubiq_cyt_C_chap ; YaaW family
2.	128912-128004	ATH66629.1	Membrane protein	DUF3300
3.	134568-134221	ATH66635.1	YaaC like Chaperone lipoprotein;	PulS_OutS; T2SS_PulS_OutS
4.	142577-143806	ATH66643.1	Polysaccharide deacetylase family protein; Putative xylanase/chitin deacetylase	CE4_yadE_5s;deacetyl_PgaB;CDA1;Polysacc_deac_1;hms F
5.	168637-168014	ATH66665.1	TRIC cation channel family protein	PRK10578; YadS; UPF0126
6.	204589-205134	ATH66697.1	Trancription elongation protein; Nucleic acid binding protein;	YaeQ
7.	207256-206432	ATH66700.1	YaeF family permuted papain like enzyme; Putative lipoprotein	NLPC_P60; Spr; Peptidase_C92
8.	257524-257832	ATH66742.1	Integral inner membrane protein	DUF 2755
9.	258985-257891	ATH66743.1	Putative lipoprotein; surface-exposed outer membrane lipoprotein YaiW	DUF1615
10.	278459-277740	ATH66760.1	Fe-S protein	GlpC; PRK06259; CCG; glycerol3P_GlpC
11.	332801-332989	ATH66809.1	Protein YaiA- always immediately downstream of a	YaiA

Supplementary Table 1- Functional domain analysis of 247 HP's present *S. flexneri* 1c Y394, using four bioinformatics tools namely CDD-BLAST, Pfam, InterProScan, and SCANPROSITE.

			Shikimate kinase.	
12.	333999-334283	ATH66812.1	Pyrimidine/Purine nucleoside phosphorylase	YaiA; DUF1255
13.	375122-372969	ATH66845.1	tetratricopeptide repeat protein; YwqK family antitoxin-E; Sel1 repeat family protein	Sel1; Ywqk
14.	385847-385269	ATH66856.1	Putative Lipoprotein	YajG;Lipoprotein_16
15.	395618-395989	ATH66865.1	Competence ComEA helix-hairpin-helix repeat region domain protein	ComEA
16.	398867-397293	ATH66868.1	SgrR family transcriptional regulator	SgrR_N; PBP2_NikA_DppA_oppA_like Superfamily
17.	407052-407624	ATH66876.1	Lipoprotein; Chaperone lipoprotein for Oligomerisation	YscW; YbaY
18.	412362-411892	ATH66880.1	Inner Membrane protein YlaC	YIaC family
19.	441081-440287	ATH66907.1	Ligase; gumN family protein	TraB; MopB_Thiosulfate-R-like family
20.	447759-447301	ATH66913.1	NfeD family protein (Nodulation efficiency protein)	NfeD; CobS; YbbJ
21.	474791-475309	ATH66937.1	Fimbrial biogenesis outer membrane usher protein precursor	PapC_N; FimD;PRK15198;PapC_N; Usher Family
22.	478463-477573	ATH66941.1	DUF4434 family protein; AmyAc_family superfamily - Alpha Amylase	DUF4434

Supplementary Table 1- Functional domain analysis of 247 HP's present *S. flexneri* 1c Y394, using four bioinformatics tools namely CDD-BLAST, Pfam, InterProScan, and SCANPROSITE.

23.	501451-501083	ATH66955.1	MmcQ/YjbR family DNA-binding protein	YjbR
24.	531057-531254	ATH66978.1	YbdD/YjiX family protein; Putative selenoprotein	YbdD; Sel_put
25.	603063-605168	ATH67045.1	Putative Fimbrial biogenesis outer membrane usher protein	FimD fam; Usher fam
26.	606337-606987	ATH67048.1	Putative fimbrial-like adhesin protein; Type 1 fimbrial protein	Fimbrial fam and superfam
27.	611315-610659	ATH67052.1	5-oxoprolinase subunit PxpB; Putative carboxylase	CT_C_D superfamily
28.	641178-640852	ATH67074.1	Putative lipoprotein; YbfN-like lipofamily protein	YbfN family
29.	670249-669542	ATH67096.1	DUF1266 domain-containing protein; Putative trna ligase	DUF1266
30.	673046-672339	ATH67099.1	DUF1266 domain-containing protein; Putative trna ligase - shigella	DUF1266
31.	673210-674187	ATH67100.1	Sel1 family TPR-like repeat protein YbeQ	Sel1 superfamily
32.	687116-687379	ATH67113.1	DUF493 family protein; Proposed lipoate regulatory protein YbeD	DUF493
33.	734084-734506	ATH67162.1	YdaT_toxin Protein	YdaT_toxin
34.	737140-737805	ATH67165.1	Epoxyqueuosine reductase QueH	(AANH) superfamily
35.	745414-	ATH67175.1	replication protein P; toprim	Torpim_N; DnaB_C; D5_N; DnaG; Intein_splicing;

Supplementary Table 1- Functional domain analysis of 247 HP's present *S. flexneri* 1c Y394, using four bioinformatics tools namely CDD-BLAST, Pfam, InterProScan, and SCANPROSITE.

	747255		domain-containing protein; bifunctional DNA primase/helicase	Toprim_2
36.	748748-749572	ATH70531.1	replication protein P; bifunctional DNA primase/helicase	RecA-like_NTPases; P-loop_NTPase; AAA_25; GP4d_helicase
37.	751108-750845	ATH67180.1	type II toxin-antitoxin system RelE/ParE family toxin; Prophage protein Gp49	Par_E toxin; Gp 49
38.	799673-798765	ATH67230.1	Uridine diphosphate-N-acetylglucosamine-binding protein YvcK	CofD_YvcK superfamily
39.	802910-803614	ATH67236.1	Bax inhibitor-1/YccA family protein; Inner membrane protein- YbhL protein	BAX inhibitor (BI)-1/YccA-like protein family
40.	807656-808066	ATH67241.1	Putative Inner membrane protein	YbhQ
41.	875709-876086	ATH67298.1	Inner membrane protein YbjM	YbjM
42.	876533-876820	ATH67300.1	DUF1418 Membrane protein	DUF1418 superfamily; YbjC
43.	878577-879053	ATH67303.1	YbjN domain-containing protein; Putative sensory transduction regulator	YbjN
44.	883610-884098	ATH67308.1	DUF2593 family Inner membrane protein	DUF2593
45.	892694-	ATH67318.1	Heavy metal-binding domain-	YbjQ_1

Supplementary Table 1- Functional domain analysis of 247 HP's present *S. flexneri* 1c Y394, using four bioinformatics tools namely CDD-BLAST, Pfam, InterProScan, and SCANPROSITE.

	893017		containing protein	
46.	893841-894854	ATH70538.1	NAD(P)-dependent oxidoreductase; Putative nucleotide di-P-sugar epimerase or dehydratase;	Fab1; Epimerase superfamily; SDR superfamily
47.	902958-902059	ATH67325.1	L-lysine exporter LysO; Putative surface protein	DUF 340; YbjE family
48.	965544-965726	ATH67371.1	Protein YcaR; tRNA methylation protein	Trm112p-like protein superfamily
49.	966623-967516	ATH67373.1	Kinase Protein	PKc-like superfamily
50.	979384-979932	ATH70540.1	DUF882 domain-containing protein; Exported protein YcbK; tat (twin-arginine translocation)	Peptidase_M15_2
51.	1005190-1004081	ATH67401.1	6-N-hydroxylaminopurine resistance protein YcbX ; Ion binding protein.	MOSC_N; Fer2 superfamily
52.	1012486-1013049	ATH67406.1	Membrane integrity-associated transporter subunit PqiC	ABC_trans_aux superfamily
53.	1022791-1022345	ATH67413.1	YccF domain-containing Inner membrane protein	YccF superfamily
54.	1047132-1046386	ATH67436.1	Group 4 capsule (G4C) polysaccharide, YmcB protein	Caps_Synth_GfcC superfamily
55.	1081427-1081918	ATH67468.1	DUF1097 domain-containing protein Inner membrane protein YcdZ; Putative Amino acid transporter	DUF1079

Supplementary Table 1- Functional domain analysis of 247 HP’s present *S. flexneri* 1c Y394, using four bioinformatics tools namely CDD-BLAST, Pfam, InterProScan, and SCANPROSITE.

56.	1155999-1156376	ATH67536.1	DUF1425 domain-containing protein; putative periplasmic lipoprotein	DUF1425 superfam; YcfL fam
57.	1158896-1159438	ATH67540.1	Alpha/beta hydrolase	Abhydrolase superfamily
58.	1168941-1167868	ATH67546.1	Putative acyltransferase family protein	Acyl_transf_3; DUF418 superfamily
59.	1218563-1219024	ATH67604.1	Putative cytoplasmic protein YcgN; YcgN family cysteine cluster protein	CxxCxxCC superfamily
60.	1258534-1258812	ATH67634.1	Stress-induced protein YchH	DUF2583 superfamily
61.	1272263-1271910	ATH67647.1	DsrE/F sulfur relay family protein YchN; Putative ACR involved in intracellular sulfur reduction	DrsE superfamily
62.	1309262-1308966	ATH67677.1	YciI family protein; Putative cytoplasmic protein YciI	YciI superfamily
63.	1314420-1314241	ATH67684.1	Putative cytoplasmic protein; YciG stress induced protein.	GsiB superfamily; KGG superfamily
64.	1348093-1347740	ATH67716.1	DUF559 domain-containing protein; Restriction endonuclease	Restriction_endonuclease_like superfamily
65.	1378871-1379551	ATH67743.1	Monomeric porin OmpG; outer membrane porin OmpG	Porin_OmpG
66.	1379576-1379743	ATH67744.1	Outer membrane porin OmpG domain protein; OmpG prot	Porin_OmpG

Supplementary Table 1- Functional domain analysis of 247 HP's present *S. flexneri* 1c Y394, using four bioinformatics tools namely CDD-BLAST, Pfam, InterProScan, and SCANPROSITE.

			precurssor protein	
67.	1381008-1382405	ATH67746.1	YcjX family protein; Putative ATP-binding protein	DUF463
68.	1444751-1444512	ATH67804.1	Membrane protein; YobH-like family protein	YobH-like family protein
69.	1448042-1447422	ATH67810.1	Manganese efflux pump MntP; Putative YebN family transport protein	Mntp
70.	1461081-1460737	ATH70565.1	RidA family protein; RutC family protein yoaB; putative translation initiation inhibitor, yjgF family; endoribonuclease L-PSP	YjgF_YER057c_UK114_like_2 superfamily
71.	1463916-1464497	ATH67825.1	Slp family lipoprotein YeaY	Slp superfamily
72.	1470396-1470494	ATH67828.1	Putative transcriptional regulator	HTH_XRE superfamily
73.	1495060-1496100	ATH67852.1	DUF1266 domain-containing protein; Inner membrane protein YnjI	DUF1266
74.	1498524-1499144	ATH67855.1	CDP-alcohol phosphatidyltransferase family protein	CDP-alcohol phosphatidyltransferase superfamily
75.	1531832-1532368	ATH67883.1	Integral membrane protein	YniB superfamily
76.	1533665-1533375	ATH67885.1	Endoribonuclease GhoS	GhoS superfamily

Supplementary Table 1- Functional domain analysis of 247 HP's present *S. flexneri* 1c Y394, using four bioinformatics tools namely CDD-BLAST, Pfam, InterProScan, and SCANPROSITE.

77.	1535759-1536517	ATH67887.1	DUF481 domain-containing protein; Salt/Acid induced outer membrane protein	DUF481
78.	1573122-1573478	ATH67921.1	RusA family crossover junction endodeoxyribonuclease; Holliday junction resolvase	RusA
79.	1585388-1584870	ATH67934.1	2-oxo-tetronate isomerase	CinA
80.	1601251-1602165	ATH67952.1	Putative membrane protein; bestrophin- RFP-TM; Chloride channel family protein	Bestrophin superfamily
81.	1606541-1606254	ATH67957.1	Autoinducer 2 ABC transporter substrate-binding protein	XlyF
82.	1616723-1616397	ATH67966.1	YnfA family protein; Putative membrane spanning protein; Putative inner membrane lipoprotein;	UPF0060
83.	1616858-1617199	ATH67967.1	DUF1283 family protein; YnfB lipoprotein	DUF1283
84.	1618615-1618920	ATH67969.1	DUF1161 domain-containing protein; YnfD lipoprotein	DUF1161
85.	1648406-1648071	ATH67997.1	GlpM family protein; Inner membrane protein associated with alginate biosynthesis	GlpM
86.	1689668-1690135	ATH68032.1	Outer membrane lipoprotein SlyB	Rick_17kDA_anti superfamily
87.	1717506-	ATH68055.1	Autotransporter;	AIDA superfamily

Supplementary Table 1- Functional domain analysis of 247 HP's present *S. flexneri* 1c Y394, using four bioinformatics tools namely CDD-BLAST, Pfam, InterProScan, and SCANPROSITE.

	1716250		Autotransporter adhesin-related protein	
88.	1723103-1722456	ATH68061.1	YdhW family putative oxidoreductase system protein	PRK09947 superfamily
89.	1725855-1725229	ATH68062.1	ferredoxin-like protein;	DMSOR_beta_like superfamily
90.	1726520-1726311	ATH68063.1	fumarate hydratase FumD; Sulfur acceptor that activates SufS cysteine desulfurase	DUF2767
91.	1740839-1737783	ATH68075.1	FAD-binding oxidoreductase	CCG superfam; FAD- oxidase_C superfamily
92.	1741228-1742340	ATH68076.1	AI-2E family transporter YdiK	AI-2E family transporter superfamily
93.	1742769-1743125	ATH68077.1	DUF1870 family protein-putative DNA binding protein	DUF1870 superfamily
94.	1761937-1763256	ATH68089.1	Glycoside hydrolase family 10 protein	YddW superfamily
95.	1796710-1797159	ATH68112.1	DMT family transporter - drug/ metabolite transporter	EamA superfamily
96.	1797393-1797160	ATH68113.1	DUF2526 domain-containing protein; putative cytoplasmic protein; Extracellular solute-binding protein	DUF2526
97.	1797700-1797479	ATH70578.1	GhoT/OrtT family toxin; YdcX inner membrane protein	Toxin_GhoT_OrtT domain-containing protein family
98.	1809312-1810154	ATH68125.1	Acetyltransferase; Putative transferase; LbetaH domain-	LbetaH superfamily

Supplementary Table 1- Functional domain analysis of 247 HP's present *S. flexneri* 1c Y394, using four bioinformatics tools namely CDD-BLAST, Pfam, InterProScan, and SCANPROSITE.

			containing protein	
99.	1826138-1825638	ATH68140.1	YdcF family protein; Protein binding with S-adenosyl-L-methionine (AdoMet).	YdcF - like superfamily
100.	1837928-1836171	ATH68145.1	alpha/beta fold hydrolase; Putative hydrolase	Abhydrolase_1; AdoMet_MTases superfamily
101.	1839444-1838839	ATH68146.1	CDP-alcohol phosphatidyltransferase family protein	CDP-alcohol phosphatidyltransferase
102.	1848256-1848582	ATH68152.1	DUF1318 domain-containing protein; YnbE family lipoprotein	DUF1318
103.	1850130-1849864	ATH68154.1	Putative hemolysin; DUF333 domain-containing protein	DUF 333; RlpA superfamily
104.	1889630-1889256	ATH68182.1	CopC domain-containing protein YobA- copper ion transport and copper ion response	Big_5 superfamily
105.	1895052-1894696	ATH68188.1	Protein YebF- colicin immunity protein.	YebF superfamily
106.	1895409-1895287	ATH68190.1	DNA damage-inducible protein YebG	YebG superfamily
107.	1910912-1911514	ATH68203.1	YebB family permuted papain-like enzyme	NLPC_P60
108.	1939210-1938851	ATH68230.1	RusA family crossover junction endodeoxyribonuclease	RusA
109.	1942685-1943254	ATH68234.1	DNA polymerase III beta subunit, central domain protein	beta_clamp superfamily
110.	1945349-	ATH68239.1	DUF72 domain-containing	DUF72

Supplementary Table 1- Functional domain analysis of 247 HP's present *S. flexneri* 1c Y394, using four bioinformatics tools namely CDD-BLAST, Pfam, InterProScan, and SCANPROSITE.

	1945699		protein; Inner membrane protein yecN; Putative cytosolic protein	
111.	1945752-1946147	ATH68240.1	Inner membrane protein YecN;	MAPEG superfamily
112.	1955458-1955946	ATH68248.1	Lysozyme inhibitor LprI family protein	DUF1311; YecT family
113.	1961217-1961675	ATH68250.1	dNTP triphosphohydrolase	YdhJ; HDc superfamily
114.	1986617-1986940	ATH68272.1	Putative Lipoprotein	YecR
115.	2006346-2005933	ATH68293.1	YedD lipoprotein	YedD
116.	2006533-2007738	ATH68294.1	Selenium metabolism membrane protein YedE/FdhT	Sulf_transp superfamily
117.	2013934-2013383	ATH68297.1	Raf kinase inhibitor-like protein YbcL; Kinase inhibitor	PEPB superfamily
118.	2027908-2028309	ATH68320.1	HAD-IIB family hydrolase; mannosyl-3-phosphoglycerate phosphatase; mannosyl-3-phosphoglycerate phosphatase-related protein	YedP; HAD-like superfamily
119.	2035144-2034947	ATH68327.1	Permease; Inner membrane protein YedR	djlA
120.	2053556-2053191	ATH68340.1	RusA family crossover junction endodeoxyribonuclease	RusA
121.	2079232-	ATH68357.1	RusA family crossover junction	RusA

Supplementary Table 1- Functional domain analysis of 247 HP's present *S. flexneri* 1c Y394, using four bioinformatics tools namely CDD-BLAST, Pfam, InterProScan, and SCANPROSITE.

	2078867		endodeoxyribonuclease	
122.	2096882-2096655	ATH68374.1	SirA family protein; Sulfurtransferase TusA	TusA
123.	2116885-2116394	ATH70596.1	Glycosyltransferase; Putative dolichol-phosphate mannosyltransferase; protein rfbJ;	Glyco_tranf_GTA-type superfamily
124.	2155614-2156063	ATH68421.1	Diguanylate cyclase; putative sensor protein; PAS domain S-box protein	COG5001
125.	2203928-2203404	ATH68460.1	Type 1 fimbrial protein; YehD protein; fimbrial-like adhesin protein	Fimbrial superfamily
126.	2222052-2223188	ATH68471.1	Mg-chelatase subunit ChlD; VWA domain-containing protein	beta_Kdo_transferase; VWA_YIEM_type domain-containing protein
127.	2223185-2225188	ATH68472.1	Peptidase	Peptidase family C69
128.	2226089-2226550	ATH68473.1	DUF1307 domain-containing protein; lipoprotein	DUF1307; YehR
129.	2256512-2256910	ATH68497.1	Putative effector of murein hydrolase LrgA; antiholin-like protein LrgA; LrgA family protein	LrgA
130.	2275278-2276327	ATH68515.1	YeiH family protein; YeiH family putative sulfate export transporter	Cons_hypoth698
131.	2295033-2295746	ATH68531.1	Kdo(2)-lipid A phosphotransferase; PAP2	PgpB

Supplementary Table 1- Functional domain analysis of 247 HP's present *S. flexneri* 1c Y394, using four bioinformatics tools namely CDD-BLAST, Pfam, InterProScan, and SCANPROSITE.

			family protein	
132.	2318104-2318313	ATH68551.1	Single-stranded DNA-binding protein	Ssb; Asp_Lys-Asn_RS_N superfamily
133.	2383925-2383659	ATH68606.1	Adhesin AIDA-I family autotransporter; Putative outer membrane autotransporter	AidA sperfamily
134.	2389228-2388578	ATH68609.1	Lipopolysaccharide kinase InaA; pH-inducible protein involved in stress response (putative kinase)	PKc_like superfamily
135.	2389443-2389649	ATH68611.1	yfaH protein; LysR family transcriptional regulator	PBP2_LTTR-Substrate
136.	2400007-2399465	ATH68620.1	YfaZ superfamily; Membrane protein	YfaZ
137.	2443638-2443144	ATH68658.1	YfbU family protein; ligand binding protein	YfbU
138.	2444176-2443721	ATH68659.1	DUF412 domain-containing protein membrane protein	DUF412
139.	2448125-2449645	ATH68662.1	putative S-transferase; putative basic amino acid antiporter YfcC; C4-dicarboxylate anaerobic carrier protein	ArsB_NhaD_permease
140.	2465785-2465126	ATH68680.1	tRNA pseudouridine(38-40) synthase TruA	PseudoU_synth
141.	2476615-2475806	ATH68691.1	Uncharacterized membrane protein YfcA	TauE
142.	2497196-2498128	ATH68709.1	Inner membrane protein YfdC; transporter protein	Form_nir_trans_superfamily

Supplementary Table 1- Functional domain analysis of 247 HP's present *S. flexneri* 1c Y394, using four bioinformatics tools namely CDD-BLAST, Pfam, InterProScan, and SCANPROSITE.

143.	2503563-2503793	ATH68713.1	Permease DsdX; D-serine transporter DsdX ; GntP family permease	GntP permease
144.	2536652-2536978	ATH68737.1	DUF2502 domain-containing protein; RNA recognition motif (RRM) domain	DUF2502
145.	2542660-2543004	ATH68741.1	putative DNA-binding transcriptional regulator; helix-turn-helix family protein	DUF1323; HTH_merR_SF
146.	2543006-2543398	ATH68742.1	putative DNA-binding transcriptional regulator; helix-turn-helix family protein	DUF1323; HTH_merR_SF
147.	2548236-2548018	ATH68747.1	putative cytoplasmic protein; DNA polymerase III subunit epsilon	PolC; QSregVF_b
148.	2557418-2557792	ATH68756.1	Trna synthetase	t-RNA-synt_2
149.	2570438-2569989	ATH68766.1	DUF2919 domain-containing inner membrane protein	DUF2919
150.	2576465-2577085	ATH70611.1	Transcriptional regulator	HATPase_c_4
151.	2580480-2580773	ATH68777.1	Glutamate decarboxylase beta domain protein	AAT_I
152.	2603582-2603782	ATH68793.1	YpfN family protein; Putative inner membrane prot	UPF0370
153.	2641931-2642449	ATH68830.1	glycine zipper 2TM domain-containing protein; membrane	Rick_17kDa_Anti superfamily

Supplementary Table 1- Functional domain analysis of 247 HP's present *S. flexneri* 1c Y394, using four bioinformatics tools namely CDD-BLAST, Pfam, InterProScan, and SCANPROSITE.

			protein	
154.	2651424-2650804	ATH68837.1	tetratricopeptide repeat protein	TPR_21
155.	2671784-2672638	ATH68856.1	alpha/beta hydrolase; peptidase	FrsA; Abhydrolase superfamily
156.	2680750-2681172	ATH68864.1	DoxX family protein; inner membrane protein YphA	DoxX superfamily
157.	2743716-2744039	ATH68917.1	YfiM family lipoprotein	DUF2279
158.	2763740-2764105	ATH68929.1	DUF2799 domain-containing protein; putative exonuclease ATPase subunit; lipoprotein	DUF2799
159.	2766004-2766486	ATH68931.1	OmpA family protein	OmpA_C- like superfamily
160.	2782729-2784981	ATH68947.1	Putative alpha amylase	Alpha-amylase superfamily
161.	2795188-2795712	ATH68957.1	thiosulfate sulfurtransferase YgaP; Inner membrane protein with hydrolase activity	ThiF; RHOD; DUF2892
162.	2797814-2798044	ATH68961.1	DUF883 domain-containing protein- Membrane-anchored ribosome-binding protein	DUF883
163.	2814611-2814183	ATH68975.1	DedA family inner membrane protein	DedA family
164.	2869170-2868847	ATH69025.1	DUF3561 family protein; Putative cytochrome oxidase subunit	DUF3561

Supplementary Table 1- Functional domain analysis of 247 HP's present *S. flexneri* 1c Y394, using four bioinformatics tools namely CDD-BLAST, Pfam, InterProScan, and SCANPROSITE.

165.	2906794-2906465	ATH69052.1	YqcC family protein- tRNA pseudouridine synthase C	DUF446
166.	2924914-2924519	ATH69068.1	DUF423 domain-containing inner membrane protein	DUF423
167.	2979755-2979000	ATH69109.1	LysM peptidoglycan-binding domain-containing protein; lipoprotein YgeR	Peptidase_M23
168.	2995934-2995527	ATH69124.1	Membrane protein YgfX	CptA_toxin
169.	3055411-3055977	ATH69181.1	Osmotic shock tolerance protein YggT ; integral membrane protein	YGGT
170.	3060596-3061885	ATH69187.1	TRAP transporter, DctM subunit	DctM; ArsB_NhaD_permease
171.	3084630-3084226	ATH69209.1	Protein glcG	Haem_degrading superfamily
172.	3116470-3116727	ATH69239.1	lipoprotein YqhH	GNVR superfam; LPP superfamily
173.	3136059-3136730	ATH69259.1	DUF1190 family protein; putative inner membrane protein	DUF1190
174.	3141094-3141444	ATH69265.1	ubiquinone biosynthesis accessory factor UbiK	PKD_channel; BMFP superfamily
175.	3188711-3189403	ATH69300.1	vancomycin high temperature exclusion protein	YdcF-like superfamily
176.	3190751-3191716	ATH69301.1	TerC family membrane protein Alx; putative pH-induced	TerC

Supplementary Table 1- Functional domain analysis of 247 HP's present *S. flexneri* 1c Y394, using four bioinformatics tools namely CDD-BLAST, Pfam, InterProScan, and SCANPROSITE.

			membrane-bound redox modulator	
177.	3207491-3207796	ATH69313.1	DUF883 domain-containing protein; membrane-anchored ribosome-binding protein; putative membrane protein YqjD	DUF 883
178.	3207799-3208203	ATH69314.1	inner membrane protein YqjE	YqjE family; Phage_holin_3_6
179.	3214309-3212999	ATH69322.1	serine dehydratase subunit alpha family protein	SDH_alpha superfamily
180.	3215668-3214337	ATH69323.1	HAAAP family serine/threonine permease; inner membrane transport protein yhaO	SLC5-6-like_sbd; stp family
181.	3257093-3256458	ATH69359.1	NAD-dependent epimerase/dehydratase family protein	SDR superfamily
182.	3258213-3258515	ATH69360.1	DNA damage response exodeoxyribonuclease YhbQ; GIY-YIG nuclease superfamily protein	GIY_YIG_Sf superfamily
183.	3306661-3307293	ATH69408.1	PhoP regulatory network protein YrbL	Pkinase
184.	3338987-3338715	ATH69432.1	Barnase inhibitor yhcO	Barstar like superfamily
185.	3359953-3360195	ATH69450.1	DUF997 family protein; Inner membrane protein	DUF997

Supplementary Table 1- Functional domain analysis of 247 HP's present *S. flexneri* 1c Y394, using four bioinformatics tools namely CDD-BLAST, Pfam, InterProScan, and SCANPROSITE.

186.	3410714-3409992	ATH69450.1	Transcritptional regulator	PAS_6
187.	3413066-3412866	ATH69519.1	YheV family putative metal-binding protein	DUF2387
188.	3420024-3419620	ATH69527.1	OsmC family protein	OsmC
189.	3420997-3423099	ATH69530.1	Integral Membrane protein	FUSC-like superfamily
190.	3441559-3440255	ATH69548.1	Integral Membrane protein	YhfT superfamily
191.	3445200-3444037	ATH69552.1	YhfX family PLP-dependent enzyme; Alanine racemase, N-terminal domain protein	Ala_racemase_C; PLPDE_3
192.	3445663-3446568	ATH69554.1	Substrate binding domain for transcriptional regulator; Periplasmic binding protein	PBP2_LTTR_substrate superfamily
193.	3466921-3466790	ATH69573.1	Endoglucanase Acf2	Glyco_hydro_81; Acf2
194.	3479710-3479979	ATH69585.1	AbrB/MazE/SpoVT family DNA-binding domain-containing protein; Antitoxin E	MazE_antitoxin
195.	3557848-355915	ATH69653.1	HlyD family secretion protein; HlyD family efflux transporter periplasmic adaptor subunit	HlyD
196.	3569980-3569693	ATH69662.1	Outer membrane usher protein, type 1 fimbrial synthesis -	Usher superfamily
197.	3587557-	ATH69677.1	Glutathione reductase	Pyr_redox_dim

Supplementary Table 1- Functional domain analysis of 247 HP's present *S. flexneri* 1c Y394, using four bioinformatics tools namely CDD-BLAST, Pfam, InterProScan, and SCANPROSITE.

	3587793			
198.	3591828-3592790	ATH69683.1	Permease	ArsP_1
199.	3629062-3629250	ATH69717.1	Cellulose biosynthesis protein	CBP_BcsR; mmmC
200.	3670435-3670136	ATH69741.1	Membrane protein; YsaB-like lipofamily protein	YsaB
201.	3672084-3671647	ATH69743.1	Inner membrane protein YiaB/A	YiaA superfamily
202.	3672471-3672130	ATH69744.1	Inner membrane protein YiaB-E.coli; YiaA/B two helix domain protein	YiaA superfamily
203.	3717974-3718336	ATH69782.1	YibL family ribosome-associated protein	DUF2810
204.	3718982-3719563	ATH70654.1	DUF3251 domain-containing protein; putative inner membrane lipoprotein.	DUF 3251
205.	3735337-3736296	ATH69795.1	divergent polysaccharide deacetylase family protein	Polysacc_deac_2
206.	3757405-3756737	ATH69815.1	JAB domain-containing protein; DNA repair protein RadC	RadC
207.	3763722-3764339	ATH69824.1	putative inner membrane protein; trimeric intracellular cation channel family protein	YadS; UPF0126
208.	3782926-3783969	ATH69836.1	ShiA-like protein; tetratricopeptide repeat protein	TPR_11; ANAPC3
209.	3825886-	ATH69872.1	DUF202 domain-containing	DUF202; YidH

Supplementary Table 1- Functional domain analysis of 247 HP's present *S. flexneri* 1c Y394, using four bioinformatics tools namely CDD-BLAST, Pfam, InterProScan, and SCANPROSITE.

	3825539		protein; Inner membrane protein YidH	
210.	3838868-3837591	ATH70659.1	DUF3748 domain-containing protein; putative Dipeptidyl aminopeptidases/acylaminoacyl-peptidase(Shigella); yidR protein	DUF3748; DPPIV_N
211.	3844997-3845653	ATH70660.1	Putative replicase; putative protein YidX; lipoprotein	PRK10215
212.	3865481-3866230	ATH69906.1	4'-phosphopantetheinyl transferase	Sfp
213.	3872412-3872948	ATH69913.1	Colicin E2 tolerance protein; CbrC family protein	CbrC; UPF0167
214.	3933224-3933562	ATH69954.1	macrodomain Ori organization protein MaoP; protein YifE	YifE; DUF413
215.	3981918-3982121	ATH69997.1	Putative lipoprotein	LPAM_2; Prokaryotic lipoprotein-attachment site
216.	3992061-3991645	ATH70006.1	Inner membrane protein YigG	PRK11371
217.	3993529-3993062	ATH70008.1	Thioesterase family protein	Hot_Dog; unchar_dom_1; 4HBT; PaaI_thioesterase; PaaI; PRK11688
218.	4032669-4034099	ATH70042.1	DUF945 family protein; putative GTP-binding protein	DUF945
219.	4049523-4050503	ATH70056.1	Xylose isomerase-like TIM barrel family protein	AP2Ec; AP_endonucl_2_N
220.	4064498-4065370	ATH70067.1	Virulence factor BrkB family protein	Virul_fac_BrkB

Supplementary Table 1- Functional domain analysis of 247 HP's present *S. flexneri* 1c Y394, using four bioinformatics tools namely CDD-BLAST, Pfam, InterProScan, and SCANPROSITE.

221.	4103725-4104024	ATH70101.1	DUF406 domain-containing protein; putative C-4 dicarboxylate transport protein	DUF406
222.	4118747-4118139	ATH70117.1	YiiX family permuted papain-like enzyme	NLPC_P60; Peptidase_C92; YycO
223.	4195331-4196026	ATH70671.1	DUF1481 domain-containing membrane protein	DUF1481
224.	4233545-4233387	ATH70198.1	4Fe-4S mono-cluster protein YjdI	Fer4_19; YjdI; Fer4_19
225.	4239831-4240460	ATH70203.1	DUF2238 domain-containing protein; Inner membrane protein yjdF	DUF2238
226.	4256029-4255151	ATH70213.1	Yjcz family like protein; Mutational suppressor of yhjH motility mutation,	YjcZ
227.	4262875-4262594	ATH70218.1	YjcB family membrane protein	YjcB
228.	4263304-4263621	ATH70219.1	signal transduction protein; putative EAL domain protein regulator; c-di-GMP phosphodiesterase PdeC	EAL; YjcC; CSS-motif
229.	4284544-4283855	ATH70237.1	Sel1 repeat protein; involved in signal transduction.	Sel1
230.	4296494-4296138	ATH70244.1	MmcQ/YjbR family DNA-binding protein	MmcQ/YjbR
231.	4330712-4330206	ATH70274.1	group 4 capsule (G4C) polysaccharide, YmcB	Caps_synth_GfcC

Supplementary Table 1- Functional domain analysis of 247 HP's present *S. flexneri* 1c Y394, using four bioinformatics tools namely CDD-BLAST, Pfam, InterProScan, and SCANPROSITE.

232.	4330932-4330651	ATH70275.1	group 4 capsule (G4C) polysaccharide, YmcB	Caps_synth_GfcC
233.	4331923-4331681	ATH70277.1	exopolysaccharide production YjbE family protein	YjbE
234.	4341114-4341797	ATH70286.1	YjiH family protein; nucleoside recognition family protein	YjiH; Gate superfamily
235.	4341794-4342255	ATH70287.1	Putative membrane protein; putative transporter; nucleoside recognition family protein-	SpmB; Gate superfamily
236.	4386466-4385270	ATH70326.1	DUF898 domain-containing protein; Inner membrane protein yjgN	YjgN; DUF898
237.	4407905-4408456	ATH70347.1	ribosome-associated protein	YjgA; DUF615
238.	4437881-4438426	ATH70373.1	cell division protein YtfB	OapA
239.	4460738-4460205	ATH70395.1	outer membrane lipoprotein Blc	Lipocalins
240.	4495036-4496199	ATH70424.1	Glutathionylspermidine synthase pre ATP-grasp family protein	GSP_Synth; PHA02117
241.	4498300-4498025	ATH70426.1	protease activator YjfN; putative inner membrane protein; DUF1471 domain-containing protein	DUF 1471; PRK14864
242.	4506798-4506523	ATH70436.1	DUF1471 domain-containing protein; Stress resistance	DUF1471

Supplementary Table 1- Functional domain analysis of 247 HP's present *S. flexneri* 1c Y394, using four bioinformatics tools namely CDD-BLAST, Pfam, InterProScan, and SCANPROSITE.

			protein	
243.	4512148-4511807	ATH70444.1	Endoribonuclease SymE	Sym_E toxin
244.	4521008-4520805	ATH70449.1	YbdD/YjiX family protein; Selenoprotein	Sel_put
245.	4545828-4545355	ATH70469.1	threonine/serine exporter	ThrE
246.	4546589-4545819	ATH70687.1	Inner membrane protein yjjP; threonine/serine exporter ThrE family protein; DUF 1212 protein	YjjP; ThrE
