# Supplementary material for: Functional Annotation and Curation of Hypothetical Proteins Present in A Newly Emerged Serotype 1c of Shigella flexneri: Emphasis on Selecting Targets for Virulence and Vaccine Design Studies
Source: Genes (Basel). 2020 Mar 23;11(3):340. doi: 10.3390/genes11030340 (PMC7141135; doi:10.3390/genes11030340)
Supplement: Supplementary file 1 [file genes-11-00340-s001.zip › Supplementary Table 4.pdf]

Supplementary Table 4- Virulence factor prediction of the 246 HP’s. Prediction and analysis was done using bioinformatic tools like VICMpred and VirulentPred which are based on PSI-Blast and Support Vector Machine (SVM) method.

S.No	Protein ID	VICMpred	VirulentPred
1.	ATH66527.1	Cellular process	Virulent
2.	ATH66629.1	Cellular process	Virulent
3.	ATH66635.1	Cellular process	Virulent
4.	ATH66643.1	Metabolism molecule	Virulent
5.	ATH66665.1	Metabolism molecule	Non-virulent
6.	ATH66697.1	Cellular process	Virulent
7.	ATH66700.1	Cellular process	Virulent
8.	ATH66742.1	Metabolism molecule	Non-virulent
9.	ATH66743.1	Cellular process	Virulent
10.	ATH66760.1	Metabolism molecule	Non-virulent
11.	ATH66809.1	Metabolism molecule	Virulent
12.	ATH66812.1	Cellular process	Virulent
13.	ATH66845.1	Metabolism molecule	Virulent
14.	ATH66856.1	Metabolism molecule	Non-virulent
15.	ATH66865.1	Metabolism molecule	Virulent
16.	ATH66868.1	Metabolism molecule	Virulent
17.	ATH66876.1	Metabolism molecule	Non-virulent
18.	ATH66880.1	Metabolism molecule	Non-virulent
19.	ATH66907.1	Metabolism molecule	Virulent
20.	ATH66913.1	Cellular process	Non-virulent
21.	ATH66937.1	Metabolism molecule	Virulent
22.	ATH66941.1	Virulence factor	Virulent
23.	ATH66955.1	Metabolism molecule	Virulent
24.	ATH66978.1	Metabolism molecule	Non-virulent

Supplementary Table 4- Virulence factor prediction of the 246 HP's. Prediction and analysis was done using bioinformatic tools like VICMpred and VirulentPred which are based on PSI-Blast and Support Vector Machine (SVM) method.

25.	ATH67045.1	Cellular process	Non-virulent
26.	ATH67048.1	Metabolism molecule	Virulent
27.	ATH67052.1	Metabolism molecule	Non-virulent
28.	ATH67074.1	Information and Storage	Non-virulent
29.	ATH67096.1	Metabolism molecule	Non-virulent
30.	ATH67099.1	Metabolism molecule	
31.	ATH67100.1	Cellular process	
32.	ATH67113.1	Information and Storage	
33.	ATH67162.1	Virulence factor	Virulent
34.	ATH67165.1	Cellular process	Virulent
35.	ATH67175.1	Metabolism molecule	Virulent
36.	ATH70531.1	Metabolism molecule	Non-virulent
37.	ATH67180.1	Cellular process	Non-virulent
38.	ATH67230.1	Cellular Process	Virulent
39.	ATH67236.1	Cellular process	Non-virulent
40.	ATH67241.1	Cellular process	Virulent
41.	ATH67298.1	Cellular process	Virulent
42.	ATH67300.1	Metabolism molecule	Non-virulent
43.	ATH67303.1	Metabolism molecule	Non-virulent
44.	ATH67308.1	Metabolism molecule	Virulent
45.	ATH67318.1	Cellular process	Virulent
46.	ATH70538.1	Cellular process	Non-virulent
47.	ATH67325.1	Cellular process	Non-virulent
48.	ATH67371.1	Cellular process	Virulent
49.	ATH67373.1	Cellular process	Non-virulent
50.	ATH70540.1	Metabolism Molecule	Non-virulent
51.	ATH67401.1	Metabolism Molecule	Non-virulent
52.	ATH67406.1	Metabolism Molecule	Virulent

Supplementary Table 4- Virulence factor prediction of the 246 HP's. Prediction and analysis was done using bioinformatic tools like VICMpred and VirulentPred which are based on PSI-Blast and Support Vector Machine (SVM) method.

53.	ATH67413.1	Cellular process	Non-virulent
54.	ATH67436.1	Cellular process	Non-virulent
55.	ATH67468.1	Metabolism Molecule	Virulent
56.	ATH67536.1	Metabolism Molecule	Non-virulent
57.	ATH67540.1	Metabolism Molecule	Non-virulent
58.	ATH67546.1	Cellular process	Non-virulent
59.	ATH67604.1	Cellular process	
60.	ATH67634.1	Cellular process	
61.	ATH67647.1	Cellular process	Virulent
62.	ATH67677.1	Metabolism molecule	Non-virulent
63.	ATH67684.1	Cellular process	Virulent
64.	ATH67716.1	Cellular process	Virulent
65.	ATH67743.1	Cellular process	Non-virulent
66.	ATH67744.1	Cellular process	Virulent
67.	ATH67746.1	Metabolism molecule	Non-virulent
68.	ATH67804.1	Cellular process	Non-virulent
69.	ATH67810.1	Metabolism molecule	Non-virulent
70.	ATH70565.1	Metabolism molecule	Non-virulent
71.	ATH67825.1	Cellular process	Virulent
72.	ATH67828.1	Cellular process	Non-virulent
73.	ATH67852.1	Metabolism molecule	Virulent
74.	ATH67855.1	Metabolism molecule	Non-virulent
75.	ATH67883.1	Metabolism molecule	Non-virulent
76.	ATH67885.1	Cellular process	Virulent
77.	ATH67887.1	Cellular process	Virulent
78.	ATH67921.1	Metabolism molecule	Non-virulent
79.	ATH67934.1	Cellular process	Non-virulent
80.	ATH67952.1	Cellular process	Virulent

Supplementary Table 4- Virulence factor prediction of the 246 HP's. Prediction and analysis was done using bioinformatic tools like VICMpred and VirulentPred which are based on PSI-Blast and Support Vector Machine (SVM) method.

81.	ATH67957.1	Cellular process	Non-virulent
82.	ATH67966.1	Cellular process	Virulent
83.	ATH67967.1	Cellular process	Virulent
84.	ATH67969.1	Metabolism molecule	Virulent
85.	ATH67997.1	Metabolism molecule	Non-virulent
86.	ATH68032.1	Metabolism molecule	Virulent
87.	ATH68055.1	Virulence factor	Virulent
88.	ATH68061.1	Cellular process	Non-virulent
89.	ATH68062.1	Metabolism molecule	Virulent
90.	ATH68063.1	Cellular process	Virulent
91.	ATH68075.1	Cellular process	Non-virulent
92.	ATH68076.1	Cellular process	Non-virulent
93.	ATH68077.1	Cellular process	Virulent
94.	ATH68089.1	Metabolism molecule	Virulent
95.	ATH68112.1	Cellular process	Non-virulent
96.	ATH68113.1	Cellular process	Non-virulent
97.	ATH70578.1	Metabolism molecule	Virulent
98.	ATH68125.1	Cellular process	Non-virulent
99.	ATH68140.1	Metabolism molecule	Non-virulent
100.	ATH68145.1	Metabolism molecule	Non-virulent
101.	ATH68146.1	Metabolism molecule	Non-virulent
102.	ATH68152.1	Cellular process	Virulent
103.	ATH68154.1	Metabolism molecule	Virulent
104.	ATH68182.1	Metabolism molecule	Virulent
105.	ATH68188.1	Cellular process	Non-virulent
106.	ATH68190.1	Cellular process	Virulent
107.	ATH68203.1	Metabolism molecule	Virulent
108.	ATH68230.1	Cellular process	Virulent

Supplementary Table 4- Virulence factor prediction of the 246 HP's. Prediction and analysis was done using bioinformatic tools like VICMpred and VirulentPred which are based on PSI-Blast and Support Vector Machine (SVM) method.

109.	ATH68234.1	Cellular process	Non-virulent
110.	ATH68239.1	Cellular process	Non-virulent
111.	ATH68240.1	Cellular process	Virulent
112.	ATH68248.1	Metabolism molecule	Virulent
113.	ATH68250.1	Cellular process	Non-virulent
114.	ATH68272.1	Cellular process	Virulent
115.	ATH68293.1	Cellular process	Non-virulent
116.	ATH68294.1	Metabolism molecule	Non-virulent
117.	ATH68297.1	Metabolism molecule	Virulent
118.	ATH68320.1	Cellular process	Non-virulent
119.	ATH68327.1	Metabolism molecule	Virulent
120.	ATH68340.1	Cellular process	Virulent
121.	ATH68357.1	Cellular process	Virulent
122.	ATH68374.1	Metabolism molecule	Virulent
123.	ATH70596.1	Virulence factor	Virulent
124.	ATH68421.1	Metabolism molecule	Non-virulent
125.	ATH68460.1	Cellular process	Virulent
126.	ATH68471.1	Cellular process	Non-virulent
127.	ATH68472.1	Cellular process	Virulent
128.	ATH68473.1	Metabolism molecule	Non-virulent
129.	ATH68497.1	Metabolism molecule	Non-virulent
130.	ATH68515.1	Metabolism molecule	Non-virulent
131.	ATH68531.1	Metabolism molecule	Non-virulent
132.	ATH68551.1	Cellular process	Virulent
133.	ATH68606.1	Metabolism molecule	Virulent
134.	ATH68609.1	Metabolism molecule	Virulent
135.	ATH68611.1	Virulence factor	Virulent
136.	ATH68620.1	Cellular process	Virulent

Supplementary Table 4- Virulence factor prediction of the 246 HP's. Prediction and analysis was done using bioinformatic tools like VICMpred and VirulentPred which are based on PSI-Blast and Support Vector Machine (SVM) method.

137.	ATH68658.1	Cellular process	Virulent
138.	ATH68659.1	Metabolism molecule	Non-virulent
139.	ATH68662.1	Metabolism molecule	Non-virulent
140.	ATH68680.1	Metabolism molecule	Non-virulent
141.	ATH68691.1	Cellular process	Virulent
142.	ATH68709.1	Metabolism molecule	Non-virulent
143.	ATH68713.1	Metabolism molecule	Virulent
144.	ATH68737.1	Cellular process	Virulent
145.	ATH68741.1	Cellular process	Virulent
146.	ATH68742.1	Cellular process	Virulent
147.	ATH68747.1	Cellular process	Virulent
148.	ATH68756.1	Metabolism molecule	Virulent
149.	ATH68766.1	Cellular process	Virulent
150.	ATH70611.1	Cellular process	Virulent
151.	ATH68777.1	Information and Storage	Non-virulent
152.	ATH68793.1	Metabolism molecule	Non-virulent
153.	ATH68830.1	Metabolism molecule	Non-virulent
154.	ATH68837.1	Metabolism molecule	Virulent
155.	ATH68856.1	Cellular process	Virulent
156.	ATH68864.1	Cellular process	Non-virulent
157.	ATH68917.1	Cellular process	Non-virulent
158.	ATH68929.1	Metabolism molecule	Virulent
159.	ATH68931.1	Metabolism molecule	Non-virulent
160.	ATH68947.1	Metabolism molecule	Non-virulent
161.	ATH68957.1	Metabolism molecule	Virulent
162.	ATH68961.1	Cellular process	Virulent
163.	ATH68975.1	Cellular process	Non-virulent
164.	ATH69025.1	Cellular process	Non-virulent

Supplementary Table 4- Virulence factor prediction of the 246 HP’s. Prediction and analysis was done using bioinformatic tools like VICMpred and VirulentPred which are based on PSI-Blast and Support Vector Machine (SVM) method.

165.	ATH69052.1	Cellular process	Virulent
166.	ATH69068.1	Cellular process	Non-virulent
167.	ATH69109.1	Metabolism molecule	Non-virulent
168.	ATH69124.1	Metabolism molecule	Virulent
169.	ATH69181.1	Cellular process	Non-virulent
170.	ATH69187.1	Metabolism molecule	Non-virulent
171.	ATH69209.1	Metabolism molecule	Non-virulent
172.	ATH69239.1	Cellular process	Virulent
173.	ATH69259.1	Cellular process	Non-virulent
174.	ATH69265.1	Cellular process	Virulent
175.	ATH69300.1	Metabolism molecule	Non-virulent
176.	ATH69301.1	Metabolism molecule	Non-virulent
177.	ATH69313.1	Metabolism molecule	Non-virulent
178.	ATH69314.1	Metabolism molecule	Non-virulent
179.	ATH69322.1	Cellular process	Virulent
180.	ATH69323.1	Metabolism molecule	Virulent
181.	ATH69359.1	Cellular process	Virulent
182.	ATH69360.1	Cellular process	Virulent
183.	ATH69408.1	Cellular process	Non-virulent
184.	ATH69432.1	Metabolism molecule	Non-virulent
185.	ATH69450.1	Metabolism molecule	Virulent
186.	ATH69450.1	Metabolism molecule	Non-virulent
187.	ATH69519.1	Metabolism molecule	Non-virulent
188.	ATH69527.1	Cellular process	Non-virulent
189.	ATH69530.1	Cellular process	Virulent
190.	ATH69548.1	Cellular process	Virulent
191.	ATH69552.1	Cellular process	Virulent

Supplementary Table 4- Virulence factor prediction of the 246 HP's. Prediction and analysis was done using bioinformatic tools like VICMpred and VirulentPred which are based on PSI-Blast and Support Vector Machine (SVM) method.

192.	ATH69554.1	Cellular process	Virulent
193.	ATH69573.1	Cellular process	Non-virulent
194.	ATH69585.1	Cellular process	Non-virulent
195.	ATH69653.1	Information and Storage	Non-virulent
196.	ATH69662.1	Metabolism molecule	Non-virulent
197.	ATH69677.1	Metabolism molecule	Non-virulent
198.	ATH69683.1	Metabolism molecule	Virulent
199.	ATH69717.1	Metabolism molecule	Non-virulent
200.	ATH69741.1	Metabolism molecule	Non-virulent
201.	ATH69743.1	Cellular process	Non-virulent
202.	ATH69744.1	Cellular process	Virulent
203.	ATH69782.1	Cellular process	Virulent
204.	ATH70654.1	Cellular process	Virulent
205.	ATH69795.1	Cellular process	Virulent
206.	ATH69815.1	Cellular process	Non-virulent
207.	ATH69824.1	Cellular process	Non-virulent
208.	ATH69836.1	Metabolism molecule	Non-virulent
209.	ATH69872.1	Cellular process	Virulent
210.	ATH70659.1	Cellular process	Non-virulent
211.	ATH70660.1	Cellular process	Virulent
212.	ATH69906.1	Metabolism molecule	Non-virulent
213.	ATH69913.1	Metabolism molecule	Non-virulent
214.	ATH69954.1	Cellular process	Non-virulent
215.	ATH69997.1	Cellular process	Virulent
216.	ATH70006.1	Cellular process	Non-virulent
217.	ATH70008.1	Cellular process	Non-virulent
218.	ATH70042.1	Cellular process	Non-virulent
219.	ATH70056.1	Cellular process	Virulent

Supplementary Table 4- Virulence factor prediction of the 246 HP's. Prediction and analysis was done using bioinformatic tools like VICMpred and VirulentPred which are based on PSI-Blast and Support Vector Machine (SVM) method.

220.	ATH70067.1	Cellular process	Virulent
221.	ATH70101.1	Cellular process	Non-virulent
222.	ATH70117.1	Metabolism molecule	Non-virulent
223.	ATH70671.1	Cellular process	Virulent
224.	ATH70198.1	Cellular process	Non-virulent
225.	ATH70203.1	Cellular process	Non-virulent
226.	ATH70213.1	Metabolism molecule	Non-virulent
227.	ATH70218.1	Metabolism molecule	Virulent
228.	ATH70219.1	Cellular process	Virulent
229.	ATH70237.1	Cellular process	Virulent
230.	ATH70244.1	Cellular process	Non-virulent
231.	ATH70274.1	Virulence factor	Virulent
232.	ATH70275.1	Virulence factor	Virulent
233.	ATH70277.1	Cellular process	Non-virulent
234.	ATH70286.1	Cellular process	Virulent
235.	ATH70287.1	Cellular process	Non-virulent
236.	ATH70326.1	Cellular process	Non-virulent
237.	ATH70347.1	Cellular process	Non-virulent
238.	ATH70373.1	Cellular process	Virulent
239.	ATH70395.1	Cellular process	Non-virulent
240.	ATH70424.1	Cellular process	Non-virulent
241.	ATH70426.1	Cellular process	Virulent
242.	ATH70436.1	Cellular process	Virulent
243.	ATH70444.1	Cellular process	Virulent
244.	ATH70449.1	Cellular process	Virulent
245.	ATH70469.1	Cellular process	Non-virulent
246.	ATH70687.1	Cellular process	Non-virulent
