# Supplementary material for: Functional Annotation and Curation of Hypothetical Proteins Present in A Newly Emerged Serotype 1c of Shigella flexneri: Emphasis on Selecting Targets for Virulence and Vaccine Design Studies
Source: Genes (Basel). 2020 Mar 23;11(3):340. doi: 10.3390/genes11030340 (PMC7141135; doi:10.3390/genes11030340)
Supplement: Supplementary file 1 [file genes-11-00340-s001.zip › Supplementary Table 2.pdf]

Supplementary Table 2- Analysis of physiochemical characteristics for the 246 HP's present in *S.flexneri* 1c Y394. ExPASy's ProtParam tool was used to study physiochemical properties of the HP's which included number of amino acids, molecular weight, theoretical pI, extinction coefficient, and grand average of hydropathicity (GRAVY).

S.No	Protein ID	No of amino acids	MW	PI	Extinction Coefficient	Grand Average of Hydrophobicity (GRAVY)
**1.**	ATH66527.1	237	26708.94	9.37	15595	-0.284
**2.**	ATH66629.1	302	34186.18	9.11	22960	-0.299
**3.**	ATH66635.1	115	12509.10	6.75	7115	-0.357
**4.**	ATH66643.1	409	46351.22	9.84	59820	-0.284
**5.**	ATH66665.1	207	22289.85	7.80	29575	0.904
**6.**	ATH66697.1	181	20876.67	4.94	50085	-0.357
**7.**	ATH66700.1	274	29970.40	7.62	24325	-0.016
**8.**	ATH66742.1	102	11396.45	9.89	21095	0.501
**9.**	ATH66743.1	364	40443.32	9.61	51005	-0.384
**10.**	ATH66760.1	239	26104.41	6.82	14940	0.026
**11.**	ATH66809.1	62	7152.09	6.72	11460	-1.239
**12.**	ATH66812.1	94	10234.40	4.47	12950	-0.039
**13.**	ATH66845.1	717	81623.66	6.06	139845	-0.823
**14.**	ATH66856.1	192	20906.02	9.04	7450	-0.062

Supplementary Table 2- Analysis of physiochemical characteristics for the 246 HP's present in *S.flexneri* 1c Y394. ExPASy's ProtParam tool was used to study physiochemical properties of the HP's which included number of amino acids, molecular weight, theoretical pI, extinction coefficient, and grand average of hydropathicity (GRAVY).

**15.**	ATH66865.1	123	12703.65	8.76	2980	0.019
**16.**	ATH66868.1	524	60063.95	6.51	109235	-0.256
**17.**	ATH66876.1	190	19441.24	7.87	6990	0.172
**18.**	ATH66880.1	156	18267.19	6.74	21430	0.103
**19.**	ATH66907.1	264	29902.48	6.24	48930	-0.228
**20.**	ATH66913.1	152	16938.82	7.07	56490	0.376
**21.**	ATH66937.1	172	19064.66	4.89	20190	-0.035
**22.**	ATH66941.1	296	33145.90	8.75	59025	-0.158
**23.**	ATH66955.1	122	14040.28	8.71	20970	-0.358
**24.**	ATH66978.1	65	7467.46	6.70	7575	-0.797
**25.**	ATH67045.1	701	77354.73	4.87	146345	-0.496
**26.**	ATH67048.1	216	24527.70	5.12	22015	-0.348
**27.**	ATH67052.1	218	23986.66	5.06	36565	0.028
**28.**	ATH67074.1	108	12016.68	7.59	8730	-0.547
**29.**	ATH67096.1	235	26939.26	5.03	72100	-0.407

Supplementary Table 2- Analysis of physiochemical characteristics for the 246 HP's present in *S.flexneri* 1c Y394. ExPASy's ProtParam tool was used to study physiochemical properties of the HP's which included number of amino acids, molecular weight, theoretical pI, extinction coefficient, and grand average of hydropathicity (GRAVY).

**30.**	ATH67099.1	235	27696.14	4.97	73715	-0.496
**31.**	ATH67100.1	325	37196.09	4.91	100980	-0.633
**32.**	ATH67113.1	87	9827.35	5.50	5960	-0.166
**33.**	ATH67162.1	140	15620.86	7.95	20970	-0.309
**34.**	ATH67165.1	221	25837.34	8.07	45880	-0.691
**35.**	ATH67175.1	123	14054.13	9.00	17085	-0.238
**36.**	ATH70531.1	613	68940.90	5.49	101925	-0.450
**37.**	ATH67180.1	274	31148.59	6.67	37150	-0.455
**38.**	ATH67230.1	87	10105.81	10.07	9970	-0.663
**39.**	ATH67236.1	302	32814.94	6.00	14440	0.030
**40.**	ATH67241.1	234	25901.87	9.62	38390	0.815
**41.**	ATH67298.1	136	15464.69	10.03	36105	0.654
**42.**	ATH67300.1	125	14203.22	10.10	22375	0.751
**43.**	ATH67303.1	95	10520.70	9.39	1490	0.463
**44.**	ATH67308.1	158	17666.10	4.12	14105	0.126
**45.**	ATH67318.1	162	18518.04	9.72	33460	0.581
**46.**	ATH70538.1	107	11421.98	4.90	4470	0.102
**47.**	ATH67325.1	337	38100.69	8.97	46660	-0.297

Supplementary Table 2- Analysis of physiochemical characteristics for the 246 HP's present in *S.flexneri* 1c Y394. ExPASy's ProtParam tool was used to study physiochemical properties of the HP's which included number of amino acids, molecular weight, theoretical pI, extinction coefficient, and grand average of hydropathicity (GRAVY).

**48.**	ATH67371.1	299	32312.01	9.18	29575	1.077
**49.**	ATH67373.1	60	6854.98	4.91	7115	-0.127
**50.**	ATH70540.1	297	34487.65	6.61	80690	-0.303
**51.**	ATH67401.1	182	20354.38	10.27	15930	-0.383
**52.**	ATH67406.1	369	40597.26	7.03	40950	-0.189
**53.**	ATH67413.1	187	20634.41	5.81	48930	-0.158
**54.**	ATH67436.1	148	16275.36	9.41	34615	0.777
**55.**	ATH67468.1	214	24155.58	6.91	37595	-0.154
**56.**	ATH67536.1	163	17060.40	9.21	39210	0.948
**57.**	ATH67540.1	191	20942.50	5.57	16960	-0.436
**58.**	ATH67546.1	125	13933.06	6.96	16055	0.035
**59.**	ATH67604.1	180	21226.18	6.13	21680	-0.591
**60.**	ATH67634.1	93	10251.77	5.08	18575	-0.032
**61.**	ATH67647.1	117	12692.75	5.02	8605	0.190
**62.**	ATH67677.1	98	10602.04	5.19	11460	-0.031
**63.**	ATH67684.1	59	6032.37	9.87	As there are no Trp, Tyr or Cys in the region considered, protein should not be visible by	-1.680

Supplementary Table 2- Analysis of physiochemical characteristics for the 246 HP's present in *S.flexneri* 1c Y394. ExPASy's ProtParam tool was used to study physiochemical properties of the HP's which included number of amino acids, molecular weight, theoretical pI, extinction coefficient, and grand average of hydropathicity (GRAVY).

					UV spectrophotometry.	
**64.**	ATH67716.1	117	13959.89	9.25	26595	-0.638
**65.**	ATH67743.1	226	25958.54	4.56	52955	-0.672
**66.**	ATH67744.1	55	6428.82	4.33	12950	-0.705
**67.**	ATH67746.1	465	52795.82	8.49	71640	-0.235
**68.**	ATH67804.1	79	8586.18	9.18	10095	0.562
**69.**	ATH67810.1	206	22032.08	5.91	30480	0.743
**70.**	ATH70565.1	114	12493.23	4.96	20970	0.024
**71.**	ATH67825.1	193	20921.12	9.55	39420	-0.120
**72.**	ATH67828.1	33	3871.38	4.32	16960	-0.191
**73.**	ATH67852.1	346	40272.23	7.57	93320	-0.296
**74.**	ATH67855.1	206	22709.81	8.49	47565	0.686
**75.**	ATH67883.1	178	20337.04	6.83	22460	0.521
**76.**	ATH67885.1	96	10864.99	4.23	13980	-0.470

Supplementary Table 2- Analysis of physiochemical characteristics for the 246 HP's present in *S.flexneri* 1c Y394. ExPASy's ProtParam tool was used to study physiochemical properties of the HP's which included number of amino acids, molecular weight, theoretical pI, extinction coefficient, and grand average of hydropathicity (GRAVY).

**77.**	ATH67887.1	252	27598.27	4.99	53860	-0.502
**78.**	ATH67921.1	118	13537.84	10.05	12950	-0.281
**79.**	ATH67934.1	172	18264.75	4.74	17085	0.105
**80.**	ATH67952.1	304	34736.88	8.27	42650	0.359
**81.**	ATH67957.1	95	10047.59	8.98	2980	0.323
**82.**	ATH67966.1	108	11905.49	9.10	44710	1.152
**83.**	ATH67967.1	113	12942.69	9.15	18115	-0.761
**84.**	ATH67969.1	101	10503.72	4.74	1740	-0.149
**85.**	ATH67997.1	111	12323.10	9.51	36565	1.312
**86.**	ATH68032.1	155	15601.67	9.36	2980	0.114
**87.**	ATH68055.1	418	42880.00	4.41	20190	0.021
**88.**	ATH68061.1	215	24442.58	4.50	32110	-0.268
**89.**	ATH68062.1	208	22366.92	8.26	22095	-0.005
**90.**	ATH68063.1	69	7907.33	9.36	1490	-0.351
**91.**	ATH68075.1	1018	113233.17	6.93	101855	-0.139
**92.**	ATH68076.1	370	39841.18	8.49	78950	1.029
**93.**	ATH68077.1	118	13847.80	5.75	28210	-0.375

Supplementary Table 2- Analysis of physiochemical characteristics for the 246 HP's present in *S.flexneri* 1c Y394. ExPASy's ProtParam tool was used to study physiochemical properties of the HP's which included number of amino acids, molecular weight, theoretical pI, extinction coefficient, and grand average of hydropathicity (GRAVY).

**94.**	ATH68089.1	439	49574.34	9.25	107955	-0.464
**95.**	ATH68112.1	149	15794.96	11.35	23490	1.197
**96.**	ATH68113.1	77	8799.88	5.59	1490	-0.636
**97.**	ATH70578.1	73	8502.34	10.18	15470	0.936
**98.**	ATH68125.1	280	30680.47	4.92	37150	0.048
**99.**	ATH68140.1	166	19111.68	5.69	31970	-0.324
**100.**	ATH68145.1	585	65584.90	9.09	83895	-0.217
**101.**	ATH68146.1	201	22544.08	8.69	35200	0.821
**102.**	ATH68152.1	108	11964.87	9.59	11460	-0.225
**103.**	ATH68154.1	88	8725.99	7.58	7365	0.335
**104.**	ATH68182.1	124	13410.22	9.48	16960	-0.262
**105.**	ATH68188.1	118	12946.72	9.27	21095	-0.393
**106.**	ATH68190.1	40	4571.21	4.46	2980	-0.432

Supplementary Table 2- Analysis of physiochemical characteristics for the 246 HP's present in *S.flexneri* 1c Y394. ExPASy's ProtParam tool was used to study physiochemical properties of the HP's which included number of amino acids, molecular weight, theoretical pI, extinction coefficient, and grand average of hydropathicity (GRAVY).

**107.**	ATH68203.1	200	22674.98	7.71	36690	-0.131
**108.**	ATH68230.1	119	13650.86	9.83	11460	-0.386
**109.**	ATH68234.1	189	21371.79	9.23	7700	-0.256
**110.**	ATH68239.1	116	13261.31	8.20	17990	-0.290
**111.**	ATH68240.1	131	15226.27	9.39	38055	0.654
**112.**	ATH68248.1	162	18875.16	4.69	28670	-0.619
**113.**	ATH68250.1	152	17467.15	9.99	17990	-0.320
**114.**	ATH68272.1	107	11755.46	6.56	6210	0.277
**115.**	ATH68293.1	137	14959.21	4.87	22585	-0.242
**116.**	ATH68294.1	401	44486.93	9.98	112660	0.573
**117.**	ATH68297.1	183	19420.85	7.77	36565	-0.192

Supplementary Table 2- Analysis of physiochemical characteristics for the 246 HP's present in *S.flexneri* 1c Y394. ExPASy's ProtParam tool was used to study physiochemical properties of the HP's which included number of amino acids, molecular weight, theoretical pI, extinction coefficient, and grand average of hydropathicity (GRAVY).

**118.**	ATH68320.1	133	15126.99	5.46	30480	-0.483
**119.**	ATH68327.1	65	7206.48	10.01	20970	0.272
**120.**	ATH68340.1	121	13841.10	9.83	11460	-0.322
**121.**	ATH68357.1	121	13841.10	9.83	11460	-0.322
**122.**	ATH68374.1	75	8092.21	4.16	12615	0.151
**123.**	ATH70596.1	163	18646.13	9.59	19035	0.340
**124.**	ATH68421.1	149	16252.48	9.86	30480	0.817
**125.**	ATH68460.1	174	18301.71	4.80	7575	0.032
**126.**	ATH68471.1	378	42308.42	6.09	47690	-0.147
**127.**	ATH68472.1	667	73639.99	5.90	94390	-0.114
**128.**	ATH68473.1	153	16591.09	7.72	7450	-0.174

Supplementary Table 2- Analysis of physiochemical characteristics for the 246 HP's present in *S.flexneri* 1c Y394. ExPASy's ProtParam tool was used to study physiochemical properties of the HP's which included number of amino acids, molecular weight, theoretical pI, extinction coefficient, and grand average of hydropathicity (GRAVY).

**129.**	ATH68497.1	132	14607.71	9.15	25565	1.072
**130.**	ATH68515.1	349	36940.16	9.24	49055	0.987
**131.**	ATH68531.1	237	26761.12	9.88	42970	0.651
**132.**	ATH68551.1	69	7308.51	8.74	8605	0.043
**133.**	ATH68606.1	88	9960.62	9.61	17210	0.059
**134.**	ATH68609.1	216	25324.18	9.31	51005	-0.482
**135.**	ATH68611.1	68	7571.05	6.54	1615	0.287
**136.**	ATH68620.1	180	18611.71	4.73	28880	-0.080
**137.**	ATH68658.1	164	19562.24	5.96	31525	-0.567
**138.**	ATH68659.1	151	17198.23	9.94	33460	0.012
**139.**	ATH68662.1	506	54795.66	7.03	67045	0.746

Supplementary Table 2- Analysis of physiochemical characteristics for the 246 HP's present in *S.flexneri* 1c Y394. ExPASy's ProtParam tool was used to study physiochemical properties of the HP's which included number of amino acids, molecular weight, theoretical pI, extinction coefficient, and grand average of hydropathicity (GRAVY).

**140.**	ATH68680.1	219	24540.33	8.63	25900	0.821
**141.**	ATH68691.1	269	28673.44	9.59	21680	0.843
**142.**	ATH68709.1	310	34454.08	5.25	48470	0.147
**143.**	ATH68713.1	76	8251.81	4.32	17085	1.164
**144.**	ATH68737.1	108	13262.69	10.11	54430	-1.443
**145.**	ATH68741.1	114	12719.61	9.45	13980	-0.532
**146.**	ATH68742.1	130	14898.09	7.77	14105	-0.538
**147.**	ATH68747.1	72	8415.05	8.01	8480	-0.194
**148.**	ATH68756.1	124	13655.81	7.80	3105	-0.187
**149.**	ATH68766.1	149	17116.28	8.77	48595	0.570
**150.**	ATH70611.1	206	24037.30	8.77	30035	-0.591

Supplementary Table 2- Analysis of physiochemical characteristics for the 246 HP's present in *S.flexneri* 1c Y394. ExPASy's ProtParam tool was used to study physiochemical properties of the HP's which included number of amino acids, molecular weight, theoretical pI, extinction coefficient, and grand average of hydropathicity (GRAVY).

**151.**	ATH68777.1	337	38100.69	8.97	46660	-0.297
**152.**	ATH68793.1	299	32312.01	9.18	29575	1.077
**153.**	ATH68830.1	60	6854.98	4.91	7115	-0.127
**154.**	ATH68837.1	297	34487.65	6.61	80690	-0.303
**155.**	ATH68856.1	182	20354.38	10.27	15930	-0.383
**156.**	ATH68864.1	274	29970.40	7.62	24325	-0.016
**157.**	ATH68917.1	701	77354.73	4.87	146345	-0.496
**158.**	ATH68929.1	216	24527.70	5.12	22015	-0.348
**159.**	ATH68931.1	218	23986.66	5.06	36565	0.028
**160.**	ATH68947.1	108	12016.68	7.59	8730	-0.547
**161.**	ATH68957.1	235	26939.26	5.03	72100	-0.407

Supplementary Table 2- Analysis of physiochemical characteristics for the 246 HP's present in *S.flexneri* 1c Y394. ExPASy's ProtParam tool was used to study physiochemical properties of the HP's which included number of amino acids, molecular weight, theoretical pI, extinction coefficient, and grand average of hydropathicity (GRAVY).

**162.**	ATH68961.1	235	27696.14	4.97	73715	-0.496
**163.**	ATH68975.1	325	37196.09	4.91	100980	-0.633
**164.**	ATH69025.1	87	9827.35	5.50	5960	-0.166
**165.**	ATH69052.1	152	16938.82	7.07	56490	0.376
**166.**	ATH69068.1	172	19064.66	4.89	20190	-0.035
**167.**	ATH69109.1	296	33145.90	8.75	59025	-0.158
**168.**	ATH69124.1	122	14040.28	8.71	20970	-0.358
**169.**	ATH69181.1	65	7467.46	6.70	7575	-0.797
**170.**	ATH69187.1	701	77354.73	4.87	146345	-0.496
**171.**	ATH69209.1	216	24527.70	5.12	22015	-0.348

Supplementary Table 2- Analysis of physiochemical characteristics for the 246 HP's present in *S.flexneri* 1c Y394. ExPASy's ProtParam tool was used to study physiochemical properties of the HP's which included number of amino acids, molecular weight, theoretical pI, extinction coefficient, and grand average of hydropathicity (GRAVY).

**172.**	ATH69239.1	218	23986.66	5.06	36565	0.028
**173.**	ATH69259.1	108	12016.68	7.59	8730	-0.547
**174.**	ATH69265.1	235	26939.26	5.03	72100	-0.407
**175.**	ATH69300.1	235	27696.14	4.97	73715	-0.496
**176.**	ATH69301.1	148	16275.36	9.41	34615	0.777
**177.**	ATH69313.1	214	24155.58	6.91	37595	-0.154
**178.**	ATH69314.1	163	17060.40	9.21	39210	0.948
**179.**	ATH69322.1	701	77354.73	4.87	146345	-0.496
**180.**	ATH69323.1	216	24527.70	5.12	22015	-0.348
**181.**	ATH69359.1	218	23986.66	5.06	36565	0.028

Supplementary Table 2- Analysis of physiochemical characteristics for the 246 HP's present in *S.flexneri* 1c Y394. ExPASy's ProtParam tool was used to study physiochemical properties of the HP's which included number of amino acids, molecular weight, theoretical pI, extinction coefficient, and grand average of hydropathicity (GRAVY).

**182.**	ATH69360.1	108	12016.68	7.59	8730	-0.547
**183.**	ATH69408.1	235	26939.26	5.03	72100	-0.407
**184.**	ATH69432.1	235	27696.14	4.97	73715	-0.496
**185.**	ATH69450.1	65	7206.48	10.01	20970	0.272
**186.**	ATH69450.1	121	13841.10	9.83	11460	-0.322
**187.**	ATH69519.1	121	13841.10	9.83	11460	-0.322
**188.**	ATH69527.1	114	12493.23	4.96	20970	0.024
**189.**	ATH69530.1	193	20921.12	9.55	39420	-0.120
**190.**	ATH69548.1	33	3871.38	4.32	16960	-0.191
**191.**	ATH69552.1	346	40272.23	7.57	93320	-0.296

Supplementary Table 2- Analysis of physiochemical characteristics for the 246 HP's present in *S.flexneri* 1c Y394. ExPASy's ProtParam tool was used to study physiochemical properties of the HP's which included number of amino acids, molecular weight, theoretical pI, extinction coefficient, and grand average of hydropathicity (GRAVY).

**192.**	ATH69554.1	136	15464.69	10.03	36105	0.654
**193.**	ATH69573.1	369	40597.26	7.03	40950	-0.189
**194.**	ATH69585.1	187	20634.41	5.81	48930	-0.158
**195.**	ATH69653.1	148	16275.36	9.41	34615	0.777
**196.**	ATH69662.1	214	24155.58	6.91	37595	-0.154
**197.**	ATH69677.1	163	17060.40	9.21	39210	0.948
**198.**	ATH69683.1	191	20942.50	5.57	16960	-0.436
**199.**	ATH69717.1	125	13933.06	6.96	16055	0.035
**200.**	ATH69741.1	180	21226.18	6.13	21680	-0.591
**201.**	ATH69743.1	93	10251.77	5.08	18575	-0.032
**202.**	ATH69744.1	717	81623.66	6.06	139845	-0.823

Supplementary Table 2- Analysis of physiochemical characteristics for the 246 HP's present in *S.flexneri* 1c Y394. ExPASy's ProtParam tool was used to study physiochemical properties of the HP's which included number of amino acids, molecular weight, theoretical pI, extinction coefficient, and grand average of hydropathicity (GRAVY).

**203.**	ATH69782.1	108	11905.49	9.10	44710	1.152
**204.**	ATH70654.1	113	12942.69	9.15	18115	-0.761
**205.**	ATH69795.1	101	10503.72	4.74	1740	-0.149
**206.**	ATH69815.1	111	12323.10	9.51	36565	1.312
**207.**	ATH69824.1	155	15601.67	9.36	2980	0.114
**208.**	ATH69836.1	418	42880.00	4.41	20190	0.021
**209.**	ATH69872.1	297	34487.65	6.61	80690	-0.303
**210.**	ATH70659.1	182	20354.38	10.27	15930	-0.383
**211.**	ATH70660.1	239	26104.41	6.82	14940	0.026
**212.**	ATH69906.1	140	15620.86	7.95	20970	-0.309
**213.**	ATH69913.1	221	25837.34	8.07	45880	-0.691

Supplementary Table 2- Analysis of physiochemical characteristics for the 246 HP's present in *S.flexneri* 1c Y394. ExPASy's ProtParam tool was used to study physiochemical properties of the HP's which included number of amino acids, molecular weight, theoretical pI, extinction coefficient, and grand average of hydropathicity (GRAVY).

**214.**	ATH69954.1	123	14054.13	9.00	17085	-0.238
**215.**	ATH69997.1	613	68940.90	5.49	101925	-0.450
**216.**	ATH70006.1	274	31148.59	6.67	37150	-0.455
**217.**	ATH70008.1	87	10105.81	10.07	9970	-0.663
**218.**	ATH70042.1	302	32814.94	6.00	14440	0.030
**219.**	ATH70056.1	234	25901.87	9.62	38390	0.815
**220.**	ATH70067.1	193	20921.12	9.55	39420	-0.120
**221.**	ATH70101.1	33	3871.38	4.32	16960	-0.191
**222.**	ATH70117.1	346	40272.23	7.57	93320	-0.296
**223.**	ATH70671.1	206	22709.81	8.49	47565	0.686
**224.**	ATH70198.1	178	20337.04	6.83	22460	0.521

Supplementary Table 2- Analysis of physiochemical characteristics for the 246 HP's present in *S.flexneri* 1c Y394. ExPASy's ProtParam tool was used to study physiochemical properties of the HP's which included number of amino acids, molecular weight, theoretical pI, extinction coefficient, and grand average of hydropathicity (GRAVY).

**225.**	ATH70203.1	96	10864.99	4.23	13980	-0.470
**226.**	ATH70213.1	252	27598.27	4.99	53860	-0.502
**227.**	ATH70218.1	304	34736.88	8.27	42650	0.359
**228.**	ATH70219.1	95	10047.59	8.98	2980	0.323
**229.**	ATH70237.1	77	8799.88	5.59	1490	-0.636
**230.**	ATH70244.1	73	8502.34	10.18	15470	0.936
**231.**	ATH70274.1	280	30680.47	4.92	37150	0.048
**232.**	ATH70275.1	166	19111.68	5.69	31970	-0.324
**233.**	ATH70277.1	108	12016.68	7.59	8730	-0.547
**234.**	ATH70286.1	235	26939.26	5.03	72100	-0.407

Supplementary Table 2- Analysis of physiochemical characteristics for the 246 HP's present in *S.flexneri* 1c Y394. ExPASy's ProtParam tool was used to study physiochemical properties of the HP's which included number of amino acids, molecular weight, theoretical pI, extinction coefficient, and grand average of hydropathicity (GRAVY).

**235.**	ATH70287.1	235	27696.14	4.97	73715	-0.496
**236.**	ATH70326.1	325	37196.09	4.91	100980	-0.633
**237.**	ATH70347.1	87	9827.35	5.50	5960	-0.166
**238.**	ATH70373.1	181	20118.78	6.28	13980	-0.499
**239.**	ATH70395.1	177	19911.71	8.88	35535	- 0.194
**240.**	ATH70424.1	387	45048.67	4.61	96175	-0.443
**241.**	ATH70426.1	91	9949.13	4.18	16960	-0.141
**242.**	ATH70436.1	91	10149.35	5.09	11460	-0.153
**243.**	ATH70444.1	113	12294.12	8.64	10095	-0.168
**244.**	ATH70449.1	67	7728.79	8.60	9065	-0.945
**245.**	ATH70469.1	157	17062.55	10.12	33460	0.882

Supplementary Table 2- Analysis of physiochemical characteristics for the 246 HP's present in *S.flexneri* 1c Y394. ExPASy's ProtParam tool was used to study physiochemical properties of the HP's which included number of amino acids, molecular weight, theoretical pI, extinction coefficient, and grand average of hydropathicity (GRAVY).

**246.**	ATH70687.1	256	28000.88	8.87	32470	0.434
