# Supplementary material for: Functional Annotation and Curation of Hypothetical Proteins Present in A Newly Emerged Serotype 1c of Shigella flexneri: Emphasis on Selecting Targets for Virulence and Vaccine Design Studies
Source: Genes (Basel). 2020 Mar 23;11(3):340. doi: 10.3390/genes11030340 (PMC7141135; doi:10.3390/genes11030340)
Supplement: Supplementary file 1 [file genes-11-00340-s001.zip › Supplementary Table 3.pdf]

Supplementary Table 3- Analysis of the subcellular localization and presence of transmembrane helices present in 246 HP's. Bioinformatic tools PSORTb, CELLO and PSLpred were used for subcellular localization; HMMTOP, SOSUI and TMHMM were used identifying soluble or membrane proteins with presence or absence of transmembrane helices; SignalP 4.1 and SecretomeP 2.0 were used for predicting signal peptide and secretory proteins.

S.No	Protein ID	Sub-Cellular localisation			Signal Peptide (SignalP)	Secretory Protein (SecretomeP)	Transmembrane Helices prediction	
		CELLO	PSORTB	PSLpred			HMMTOP /TMHMM	SOSUI
1.	ATH66527.1	Cytoplasmic	Cytoplasmic	Cytoplasmic	NO	YES	NO	Soluble
2.	ATH66629.1	Cytoplasmic	Unknown	Cytoplasmic	YES	NO	NO	Soluble
3.	ATH66635.1	Extracellular	Unknown	Extracellular	NO	NO	NO	Soluble
4.	ATH66643.1	Inner membrane	Unknown	Inner membrane	YES	NO	Yes;1TM	Membrane; 1 TM
5.	ATH66665.1	Inner membrane	Cytoplasmic-Membrane	Inner membrane	NO	YES	Yes; 7TM	Membrane; 7 TM
6.	ATH66697.1	Cytoplasmic	Cytoplasmic	Cytoplasmic	NO	NO	NO	Soluble
7.	ATH66700.1	Periplasmic	Unknown	Periplasmic	YES	NO	NO	Soluble
8.	ATH66742.1	Periplasmic	Cytoplasmic-Membrane	Inner Membrane	NO	NO	Yes; 2TM	Membrane; 2 TM
9.	ATH66743.	Periplasmic	Unknown	Periplasmic	YES	NO	NO	Soluble

Supplementary Table 3- Analysis of the subcellular localization and presence of transmembrane helices present in 246 HP's. Bioinformatic tools PSORTb, CELLO and PSLpred were used for subcellular localization; HMMTOP, SOSUI and TMHMM were used identifying soluble or membrane proteins with presence or absence of transmembrane helices; SignalP 4.1 and SecretomeP 2.0 were used for predicting signal peptide and secretory proteins.

	1							
10.	ATH66760.1	Cytoplasmic	Cytoplasmic-Membrane	Cytoplasmic	NO	NO	NO	Soluble
11.	ATH66809.1	Periplasmic	Unknown	Periplasmic	NO	NO	NO	Soluble
12.	ATH66812.1	Cytoplasmic	Unknown	Cytoplasmic	NO	NO	NO	Soluble
13.	ATH66845.1	Outer Membrane	Extracellular	Extracellular	YES	NO	1TM/NO	Membrane; 1 TM
14.	ATH66856.1	Periplasmic	Unknown	Periplasmic	YES	NO	1TM/NO	Soluble
15.	ATH66865.1	Periplasmic	Cytoplasmic-Membrane	Periplasmic	YES	NO	NO	Soluble
16.	ATH66868.1	Cytoplasmic	Unknown	Cytoplasmic	NO	YES	NO	Soluble
17.	ATH66876.1	Periplasmic	Periplasmic	Periplasmic	YES	NO	NO	Soluble
18.	ATH66880.1	Inner-Membrane	Cytoplasmic-Membrane	Inner-Membrane	NO	NO	Yes; 2 TM	Membrane; 2 TM
19.	ATH66907.1	Inner membrane/Cytoplasmic	Cytoplasmic	Cytoplasmic	NO	YES	NO	Soluble

Supplementary Table 3- Analysis of the subcellular localization and presence of transmembrane helices present in 246 HP's. Bioinformatic tools PSORTb, CELLO and PSLpred were used for subcellular localization; HMMTOP, SOSUI and TMHMM were used identifying soluble or membrane proteins with presence or absence of transmembrane helices; SignalP 4.1 and SecretomeP 2.0 were used for predicting signal peptide and secretory proteins.

20.	ATH66913.1	Inner-Membrane	Cytoplasmic-Membrane	Inner-Membrane	NO	NO	Yes; 3TM	Membrane; 3 TM
21.	ATH66937.1	Outer membrane	Outer membrane	Outer membrane	YES	NO	Yes; 1TM	Membrane; 1 TM
22.	ATH66941.1	Periplasmic	Cytoplasmic-membrane	Periplasmic	YES	NO	Yes; 1TM	Membrane; 1 TM
23.	ATH66955.1	Cytoplasmic	Unknown	Cytoplasmic	NO	YES	NO	Soluble
24.	ATH66978.1	Periplasmic	Unknown	Periplasmic	NO	NO	NO	Soluble
25.	ATH67045.1	Outer membrane	Outer membrane	Outer membrane	NO	NO	NO	Soluble
26.	ATH67048.1	Outer membrane/ Cytoplasmic	Unknown	Extracellular	NO	YES	NO	Soluble
27.	ATH67052.1	Cytoplasmic	Cytoplasmic	Cytoplasmic	NO	YES	NO	Soluble
28.	ATH67074.1	Periplasmic	Unknown	Periplasmic	YES	NO	YES 1TM/NO	Soluble
29.	ATH67096.1	Cytoplasmic/ Extracellular	Cytoplasmic membrane	Cytoplasmic	YES	NO	NO	Soluble
30.	ATH67099.1	Cytoplasmic	Cytoplasmic	Cytoplasmic	NO	NO	NO	Soluble

Supplementary Table 3- Analysis of the subcellular localization and presence of transmembrane helices present in 246 HP's. Bioinformatic tools PSORTb, CELLO and PSLpred were used for subcellular localization; HMMTOP, SOSUI and TMHMM were used identifying soluble or membrane proteins with presence or absence of transmembrane helices; SignalP 4.1 and SecretomeP 2.0 were used for predicting signal peptide and secretory proteins.

31.	ATH67100.1	Outer membrane	Extracellular	Periplasmic	NO	NO	NO	Soluble
32.	ATH67113.1	Cytoplasmic	Cytoplasmic	Cytoplasmic	NO	YES	NO	Soluble
33.	ATH67162.1	Cytoplasmic	Unknown	Cytoplasmic	YES	NO	NO	Soluble
34.	ATH67165.1	Cytoplasmic	Cytoplasmic	Cytoplasmic	NO	YES	NO	Soluble
35.	ATH67175.1	Cytoplasmic	Cytoplasmic	Cytoplasmic	NO	NO	NO	Soluble
36.	ATH70531.1	Cytoplasmic	Cytoplasmic	Cytoplasmic	NO	NO	NO	Soluble
37.	ATH67180.1	Cytoplasmic	Unknown	Periplasmic	NO	NO	YES 1TM/NO	Soluble
38.	ATH67230.1	Cytoplasmic	Cytoplasmic	Cytoplasmic	YES	NO	YES 1TM/NO	Soluble
39.	ATH67236.1	Inner-membrane	Cytoplasmic membrane	Inner-membrane	NO	NO	YES; 7 TM	Membrane; 7 TM
40.	ATH67241.1	Inner-membrane	Cytoplasmic membrane	Inner-membrane	NO	YES	YES; 4TM	Membrane; 4 TM
41.	ATH67298.1	Inner-membrane	Cytoplasmic membrane	Inner-membrane	NO	NO	YES; 4TM	Membrane; 4 TM

Supplementary Table 3- Analysis of the subcellular localization and presence of transmembrane helices present in 246 HP's. Bioinformatic tools PSORTb, CELLO and PSLpred were used for subcellular localization; HMMTOP, SOSUI and TMHMM were used identifying soluble or membrane proteins with presence or absence of transmembrane helices; SignalP 4.1 and SecretomeP 2.0 were used for predicting signal peptide and secretory proteins.

42.	ATH67300.1	Inner-membrane	Cytoplasmic membrane	Inner-membrane	NO	NO	YES; 2TM	Membrane; 2 TM
43.	ATH67303.1	Cytoplasmic	Cytoplasmic membrane	Cytoplasmic	NO	NO	NO	Soluble
44.	ATH67308.1	Inner-membrane	Cytoplasmic membrane	Inner-membrane	NO	NO	YES; 4TM	Membrane; 4 TM
45.	ATH67318.1	Cytoplasmic	Unknown	Cytoplasmic	NO	NO	Yes; 1TM/No	Soluble
46.	ATH70538.1	Cytoplasmic	Cytoplasmic	Cytoplasmic	YES	NO	NO	Soluble
47.	ATH67325.1	Inner-membrane	Cytoplasmic membrane	Inner-membrane	NO	YES	Yes; 8-9 TM	Membrane; 9 TM
48.	ATH67371.1	Cytoplasmic	Unknown	Cytoplasmic	NO	NO	NO	Soluble
49.	ATH67373.1	Cytoplasmic	Cytoplasmic	Cytoplasmic	NO	NO	NO	Soluble
50.	ATH70540.1	Periplasmic	Unknown	Cytoplasmic	YES	NO	Yes; 1TM	Membrane; 1 TM
51.	ATH67401.1	Periplasmic	Unknown	Cytoplasmic	NO	NO	Yes 1TM/ NO	Soluble
52.	ATH67406.1	Outer Membrane	Unknown	Periplasmic	YES	NO	YES 1TM/NO	Soluble

Supplementary Table 3- Analysis of the subcellular localization and presence of transmembrane helices present in 246 HP's. Bioinformatic tools PSORTb, CELLO and PSLpred were used for subcellular localization; HMMTOP, SOSUI and TMHMM were used identifying soluble or membrane proteins with presence or absence of transmembrane helices; SignalP 4.1 and SecretomeP 2.0 were used for predicting signal peptide and secretory proteins.

53.	ATH67413.1	Inner-membrane	Cytoplasmic membrane	Inner-membrane	NO	NO	Yes 3 TM	Membrane; 3 TM
54.	ATH67436.1	Inner - Membrane	Cytoplasmic Membrane	Periplasmic Protein	YES	NO	NO	Soluble
55.	ATH67468.1	Inner-membrane	Cytoplasmic membrane	Inner-membrane	NO	NO	Yes; 4-5TM	Membrane; 4 TM
56.	ATH67536.1	Periplasmic	Unknown	Periplasmic	YES	NO	YES; 1TM	Membrane; 1 TM
57.	ATH67540.1	Cytoplasmic	Unknown	Cytoplasmic	YES	NO	NO	Soluble
58.	ATH67546.1	Cytoplasmic	Cytoplasmic	Cytoplasmic	NO	NO	NO	Soluble
59.	ATH67604.1	Inner-membrane	Cytoplasmic membrane	Inner-membrane	NO	NO	Yes; 8-9 TM	Membrane; 8 TM
60.	ATH67634.1	Cytoplasmic	Cytoplasmic	Cytoplasmic	NO	YES	NO	Soluble
61.	ATH67647.1	Cytoplasmic	Unknown	Cytoplasmic	NO	NO	NO	Soluble
62.	ATH67677.1	Cytoplasmic	Unknown	Cytoplasmic	NO	YES	NO	Soluble
63.	ATH67684.	Extracellular	Unknown	Extracellular	NO	YES	NO	Soluble

Supplementary Table 3- Analysis of the subcellular localization and presence of transmembrane helices present in 246 HP's. Bioinformatic tools PSORTb, CELLO and PSLpred were used for subcellular localization; HMMTOP, SOSUI and TMHMM were used identifying soluble or membrane proteins with presence or absence of transmembrane helices; SignalP 4.1 and SecretomeP 2.0 were used for predicting signal peptide and secretory proteins.

	1							
64.	ATH67716.1	Cytoplasmic	Unknown	Cytoplasmic	NO	NO	NO	Soluble
65.	ATH67743.1	Outer Membrane	Outer Membrane	Outer Membrane	NO	YES	NO	Soluble
66.	ATH67744.1	Cytoplasmic	Outer Membrane	Outer Membrane	NO	NO	NO	Soluble
67.	ATH67746.1	Cytoplasmic	Cytoplasmic	Cytoplasmic	YES	NO	NO	Soluble
68.	ATH67804.1	Inner-membrane	Cytoplasmic membrane	Inner-membrane	YES	NO	Yes; 1TM	Membrane; 1 TM
69.	ATH67810.1	Inner-membrane	Cytoplasmic membrane	Inner-membrane	NO	NO	Yes; 6 TM	Membrane; 5 TM
70.	ATH70565.1	Cytoplasmic	Cytoplasmic	Cytoplasmic	NO	NO	NO	Soluble
71.	ATH67825.1	Periplasmic	Outer Membrane	Periplasmic	NO	YES	Yes; 1TM	Soluble
72.	ATH67828.1	Cytoplasmic	Unknown	Extracellular	NO	NO	NO	Soluble
73.	ATH67852.1	Cytoplasmic	Cytoplasmic	Cytoplasmic	NO	NO	Yes; 2-3 TM	Membrane; 2 TM
74.	ATH67855.	Inner-	Cytoplasmic	Inner-membrane	NO	NO	Yes; 4-5 TM	Membrane; 5 TM

Supplementary Table 3- Analysis of the subcellular localization and presence of transmembrane helices present in 246 HP's. Bioinformatic tools PSORTb, CELLO and PSLpred were used for subcellular localization; HMMTOP, SOSUI and TMHMM were used identifying soluble or membrane proteins with presence or absence of transmembrane helices; SignalP 4.1 and SecretomeP 2.0 were used for predicting signal peptide and secretory proteins.

	1	membrane	membrane					
75.	ATH67883.1	Inner-membrane	Cytoplasmic membrane	Inner-membrane	NO	NO	Yes; 3 TM	Membrane; 3 TM
76.	ATH67885.1	Cytoplasmic	Unknown	Extracellular	NO	NO	NO	Soluble
77.	ATH67887.1	Outer Membrane	Unknown	Extracellular	YES	YES	Yes; 1TM	Membrane; 1TM
78.	ATH67921.1	Cytoplasmic	Cytoplasmic membrane	Inner-membrane	NO	NO	NO	Soluble
79.	ATH67934.1	Cytoplasmic	Unknown	Cytoplasmic	NO	NO	NO	Membrane; 1TM
80.	ATH67952.1	Inner-membrane	Cytoplasmic membrane	Inner-membrane	NO	NO	Yes; 3 TM	Membrane; 4 TM
81.	ATH67957.1	Periplasmic	Unknown	Inner-membrane	YES	NO	Yes; 1TM	Membrane; 1TM
82.	ATH67966.1	Inner-membrane	Cytoplasmic membrane	Inner-membrane	NO	NO	Yes; 4 TM	Membrane; 4 TM
83.	ATH67967.1	Periplasmic	Unknown	Periplasmic	YES	NO	Yes; 1TM	Membrane; 1TM
84.	ATH67969.1	Periplasmic	Unknown	Periplasmic	YES	YES	NO	Membrane; 1TM
85.	ATH67997.	Inner-	Cytoplasmic	Inner-membrane	NO	NO	Yes; 4 TM	Membrane; 4 TM

Supplementary Table 3- Analysis of the subcellular localization and presence of transmembrane helices present in 246 HP's. Bioinformatic tools PSORTb, CELLO and PSLpred were used for subcellular localization; HMMTOP, SOSUI and TMHMM were used identifying soluble or membrane proteins with presence or absence of transmembrane helices; SignalP 4.1 and SecretomeP 2.0 were used for predicting signal peptide and secretory proteins.

	1	membrane	membrane					
86.	ATH68032.1	Extracellular	Outer Membrane	Extracellular	YES	YES	Yes; 1TM	Membrane; 1TM
87.	ATH68055.1	Extracellular	Unknown	Cytoplasmic	NO	NO	NO	Soluble
88.	ATH68061.1	Cytoplasmic	Cytoplasmic	Cytoplasmic	NO	NO	NO	Soluble
89.	ATH68062.1	Periplasmic	Cytoplasmic	Periplasmic	YES	NO	NO	Soluble
90.	ATH68063.1	Cytoplasmic	Cytoplasmic	Cytoplasmic	NO	NO	NO	Soluble
91.	ATH68075.1	Cytoplasmic	Cytoplasmic	Cytoplasmic	NO	NO	NO	Soluble
92.	ATH68076.1	Inner-membrane	Cytoplasmic membrane	Inner-membrane	NO	YES	Yes; 9-10 TM	Membrane; 10 TM
93.	ATH68077.1	Cytoplasmic	Unknown	Cytoplasmic	NO	NO	NO	Soluble
94.	ATH68089.1	Periplasmic	Unknown	Periplasmic	YES	YES	Yes; 1TM	Membrane; 1TM
95.	ATH68112.1	Inner-membrane	Cytoplasmic membrane	Inner-membrane	NO	NO	Yes; 5 TM	Membrane; 5 TM
96.	ATH68113.	Cytoplasmic	Unknown	Cytoplasmic	NO	NO	NO	Soluble

Supplementary Table 3- Analysis of the subcellular localization and presence of transmembrane helices present in 246 HP's. Bioinformatic tools PSORTb, CELLO and PSLpred were used for subcellular localization; HMMTOP, SOSUI and TMHMM were used identifying soluble or membrane proteins with presence or absence of transmembrane helices; SignalP 4.1 and SecretomeP 2.0 were used for predicting signal peptide and secretory proteins.

	1							
97.	ATH70578.1	Inner-membrane	Cytoplasmic membrane	Inner-membrane	NO	NO	Yes; 2 TM	Membrane; 2 TM
98.	ATH68125.1	Cytoplasmic	Cytoplasmic	Cytoplasmic	NO	NO	NO	Soluble
99.	ATH68140.1	Cytoplasmic	Cytoplasmic	Cytoplasmic	NO	NO	NO	Soluble
100.	ATH68145.1	Inner-membrane	Cytoplasmic	Cytoplasmic	NO	NO	NO	Soluble
101.	ATH68146.1	Inner-membrane	Cytoplasmic membrane	Inner-membrane	NO	NO	Yes; 5-6 TM	Membrane; 5 TM
102.	ATH68152.1	Periplasmic	Unknown	Cytoplasmic	NO	YES	Yes; 1TM	Membrane; 1TM
103.	ATH68154.1	Periplasmic	Unknown	Extracellular	NO	YES	NO	Membrane; 1TM
104.	ATH68182.1	Periplasmic	Periplasmic	Periplasmic	NO	YES	NO	Soluble
105.	ATH68188.1	Periplasmic	Extracellular	Periplasmic	NO	YES	NO	Soluble
106.	ATH68190.1	Cytoplasmic	Unknown	Cytoplasmic	NO	NO	NO	Soluble
107.	ATH68203.	Cytoplasmic	Unknown	Cytoplasmic	NO	NO	NO	Soluble

Supplementary Table 3- Analysis of the subcellular localization and presence of transmembrane helices present in 246 HP's. Bioinformatic tools PSORTb, CELLO and PSLpred were used for subcellular localization; HMMTOP, SOSUI and TMHMM were used identifying soluble or membrane proteins with presence or absence of transmembrane helices; SignalP 4.1 and SecretomeP 2.0 were used for predicting signal peptide and secretory proteins.

	1							
108.	ATH68230.1	Cytoplasmic	Unknown	Cytoplasmic	NO	NO	NO	Soluble
109.	ATH68234.1	Cytoplasmic	Cytoplasmic	Cytoplasmic	NO	NO	NO	Soluble
110.	ATH68239.1	Cytoplasmic	Cytoplasmic	Periplasmic	NO	NO	NO	Soluble
111.	ATH68240.1	Inner-membrane	Cytoplasmic membrane	Inner-membrane	NO	NO	Yes; 3-4 TM	Membrane; 3 TM
112.	ATH68248.1	Cytoplasmic	Unknown	Extracellular	NO	NO	NO	Membrane; 1 TM
113.	ATH68250.1	Cytoplasmic	Cytoplasmic	Cytoplasmic	NO	NO	NO	Soluble
114.	ATH68272.1	Cytoplasmic	Unknown	Inner-membrane	NO	NO	NO	Membrane; 1 TM
115.	ATH68293.1	Periplasmic	Unknown	Periplasmic	NO	YES	NO	Membrane; 1 TM
116.	ATH68294.1	Inner-membrane	Cytoplasmic membrane	Inner-membrane	NO	NO	Yes; 10 TM	Membrane; 10 TM
117.	ATH68297.1	Periplasmic	Periplasmic	Periplasmic	NO	YES	Yes; 1 TM	Membrane; 1 TM
118.	ATH68320.	Cytoplasmic	Unknown	Cytoplasmic	NO	NO	NO	Soluble

Supplementary Table 3- Analysis of the subcellular localization and presence of transmembrane helices present in 246 HP’s. Bioinformatic tools PSORTb, CELLO and PSLpred were used for subcellular localization; HMMTOP, SOSUI and TMHMM were used identifying soluble or membrane proteins with presence or absence of transmembrane helices; SignalP 4.1 and SecretomeP 2.0 were used for predicting signal peptide and secretory proteins.

	1							
119.	ATH68327.1	Cytoplasmic	Cytoplasmic membrane	Inner-membrane	NO	NO	Yes; 1 TM	Membrane; 1 TM
120.	ATH68340.1	Cytoplasmic	Cytoplasmic	Cytoplasmic	NO	NO	NO	Soluble
121.	ATH68357.1	Cytoplasmic	Cytoplasmic	Cytoplasmic	NO	NO	NO	Soluble
122.	ATH68374.1	Cytoplasmic	Unknown	Cytoplasmic	NO	YES	NO	Soluble
123.	ATH70596.1	Cytoplasmic	Unknown	Inner-membrane	YES	NO	YES; 2TM	Membrane; 2 TM
124.	ATH68421.1	Inner-membrane	Cytoplasmic membrane	Inner-membrane	NO	NO	Yes; 5 TM	Membrane; 4 TM
125.	ATH68460.1	Extracellular	Unknown	Extracellular	NO	YES	Yes; 1 TM	Membrane; 1 TM
126.	ATH68471.1	Cytoplasmic	Cytoplasmic	Cytoplasmic	NO	NO	NO	Soluble
127.	ATH68472.1	Inner-membrane	Cytoplasmic membrane	Cytoplasmic	NO	NO	NO	Soluble
128.	ATH68473.1	Periplasmic	Unknown	Periplasmic	NO	YES	Yes; 1 TM	Membrane; 1 TM
129.	ATH68497.	Inner-	Cytoplasmic	Inner-membrane	NO	NO	Yes; 4 TM	Membrane; 3 TM

Supplementary Table 3- Analysis of the subcellular localization and presence of transmembrane helices present in 246 HP's. Bioinformatic tools PSORTb, CELLO and PSLpred were used for subcellular localization; HMMTOP, SOSUI and TMHMM were used identifying soluble or membrane proteins with presence or absence of transmembrane helices; SignalP 4.1 and SecretomeP 2.0 were used for predicting signal peptide and secretory proteins.

	1	membrane	membrane					
130.	ATH68515.1	Inner-membrane	Cytoplasmic membrane	Inner-membrane	NO	NO	Yes; 9 TM	Membrane; 10 TM
131.	ATH68531.1	Inner-membrane	Cytoplasmic membrane	Inner-membrane	NO	NO	Yes; 5 TM	Membrane; 5 TM
132.	ATH68551.1	Periplasmic	Unknown	Periplasmic	NO	YES	NO	Soluble
133.	ATH68606.1	Cytoplasmic/ Periplasmic	Unknown	Periplasmic	YES	NO	YES; 2TM	Soluble
134.	ATH68609.1	Cytoplasmic	Cytoplasmic	Cytoplasmic	NO	NO	NO	Soluble
135.	ATH68611.1	Cytoplasmic	Unknown	Cytoplasmic	NO	NO	NO	Soluble
136.	ATH68620.1	Outer-membrane	Unknown	Outer-membrane	NO	YES	Yes; 1 TM	Membrane; 1 TM
137.	ATH68658.1	Cytoplasmic	Cytoplasmic	Inner-membrane	NO	NO	NO	Soluble
138.	ATH68659.1	Inner-membrane	Cytoplasmic membrane	Inner-membrane	NO	NO	Yes; 1TM	Membrane; 1TM
139.	ATH68662.1	Inner-membrane	Cytoplasmic membrane	Inner-membrane	NO	NO	Yes; 13 TM	Membrane; 10 TM
140.	ATH68680.	Inner-	Cytoplasmic	Inner-membrane	NO	YES	Yes; 6 TM	Membrane; 5 TM

Supplementary Table 3- Analysis of the subcellular localization and presence of transmembrane helices present in 246 HP's. Bioinformatic tools PSORTb, CELLO and PSLpred were used for subcellular localization; HMMTOP, SOSUI and TMHMM were used identifying soluble or membrane proteins with presence or absence of transmembrane helices; SignalP 4.1 and SecretomeP 2.0 were used for predicting signal peptide and secretory proteins.

	1	membrane	membrane					
141.	ATH68691.1	Inner-membrane	Cytoplasmic membrane	Inner-membrane	NO	NO	Yes; 9 TM	Membrane; 7 TM
142.	ATH68709.1	Inner-membrane	Cytoplasmic membrane	Inner-membrane	NO	NO	Yes; 6 TM	Membrane; 4 TM
143.	ATH68713.1	Inner-membrane	Cytoplasmic membrane	Inner-membrane	NO	NO	Yes; 2 TM	Membrane; 2 TM
144.	ATH68737.1	Periplasmic	Unknown	Cytoplasmic	NO	NO	YES; 1TM	Soluble
145.	ATH68741.1	Cytoplasmic	Unknown	Cytoplasmic	NO	NO	NO	Soluble
146.	ATH68742.1	Cytoplasmic	Unknown	Cytoplasmic	NO	NO	NO	Soluble
147.	ATH68747.1	Cytoplasmic	Unknown	Cytoplasmic	NO	NO	NO	Soluble
148.	ATH68756.1	Cytoplasmic	Unknown	Cytoplasmic	NO	NO	NO	Membrane; 1 TM
149.	ATH68766.1	Inner-membrane	Cytoplasmic membrane	Inner-membrane	NO	NO	Yes; 4 TM	Membrane; 4 TM
150.	ATH70611.1	Cytoplasmic	Unknown	Cytoplasmic	NO	NO	NO	Soluble
151.	ATH68777.	Inner-	Cytoplasmic	Inner-membrane	NO	NO	Yes 3 TM	Membrane; 3 TM

Supplementary Table 3- Analysis of the subcellular localization and presence of transmembrane helices present in 246 HP's. Bioinformatic tools PSORTb, CELLO and PSLpred were used for subcellular localization; HMMTOP, SOSUI and TMHMM were used identifying soluble or membrane proteins with presence or absence of transmembrane helices; SignalP 4.1 and SecretomeP 2.0 were used for predicting signal peptide and secretory proteins.

	1	membrane	membrane					
152.	ATH68793.1	Inner - Membrane	Cytoplasmic Membrane	Periplasmic Protein	YES	NO	NO	Soluble
153.	ATH68830.1	Cytoplasmic	Unknown	Cytoplasmic	NO	YES	NO	Soluble
154.	ATH68837.1	Cytoplasmic	Unknown	Inner-membrane	YES	NO	YES; 2TM	Membrane; 2 TM
155.	ATH68856.1	Inner-Membrane	Cytoplasmic-Membrane	Inner-Membrane	NO	NO	Yes; 2 TM	Membrane; 2 TM
156.	ATH68864.1	Inner membrane/Cytoplasmic	Cytoplasmic	Cytoplasmic	NO	YES	NO	Soluble
157.	ATH68917.1	Inner-Membrane	Cytoplasmic-Membrane	Inner-Membrane	NO	NO	Yes; 3TM	Membrane; 3 TM
158.	ATH68929.1	Cytoplasmic	Unknown	Cytoplasmic	NO	YES	NO	Soluble
159.	ATH68931.1	Periplasmic	Periplasmic	Periplasmic	YES	NO	NO	Soluble
160.	ATH68947.1	Inner-Membrane	Cytoplasmic-Membrane	Inner-Membrane	NO	NO	Yes; 2 TM	Membrane; 2 TM
161.	ATH68957.	Cytoplasmic	Cytoplasmic	Cytoplasmic	NO	NO	NO	Soluble

Supplementary Table 3- Analysis of the subcellular localization and presence of transmembrane helices present in 246 HP's. Bioinformatic tools PSORTb, CELLO and PSLpred were used for subcellular localization; HMMTOP, SOSUI and TMHMM were used identifying soluble or membrane proteins with presence or absence of transmembrane helices; SignalP 4.1 and SecretomeP 2.0 were used for predicting signal peptide and secretory proteins.

	1							
162.	ATH68961.1	Cytoplasmic	Unknown	Cytoplasmic	NO	NO	NO	Soluble
163.	ATH68975.1	Cytoplasmic	Cytoplasmic	Cytoplasmic	NO	NO	NO	Soluble
164.	ATH69025.1	Periplasmic	Unknown	Cytoplasmic	YES	NO	Yes; 1TM	Membrane; 1 TM
165.	ATH69052.1	Periplasmic	Unknown	Cytoplasmic	NO	NO	Yes 1TM/ NO	Soluble
166.	ATH69068.1	Outer Membrane	Unknown	Periplasmic	YES	NO	YES 1TM/NO	Soluble
167.	ATH69109.1	Inner-membrane	Cytoplasmic membrane	Inner-membrane	NO	NO	Yes 3 TM	Membrane; 3 TM
168.	ATH69124.1	Cytoplasmic/ Extracellular	Cytoplasmic membrane	Cytoplasmic	YES	NO	NO	Soluble
169.	ATH69181.1	Cytoplasmic	Cytoplasmic	Cytoplasmic	NO	NO	NO	Soluble
170.	ATH69187.1	Outer membrane	Extracellular	Periplasmic	NO	NO	NO	Soluble
171.	ATH69209.1	Cytoplasmic	Unknown	Cytoplasmic	NO	YES	NO	Soluble
172.	ATH69239.	Cytoplasmic	Unknown	Inner-membrane	YES	NO	YES; 2TM	Membrane; 2 TM

Supplementary Table 3- Analysis of the subcellular localization and presence of transmembrane helices present in 246 HP's. Bioinformatic tools PSORTb, CELLO and PSLpred were used for subcellular localization; HMMTOP, SOSUI and TMHMM were used identifying soluble or membrane proteins with presence or absence of transmembrane helices; SignalP 4.1 and SecretomeP 2.0 were used for predicting signal peptide and secretory proteins.

	1							
173.	ATH69259.1	Cytoplasmic	Unknown	Cytoplasmic	NO	YES	NO	Soluble
174.	ATH69265.1	Periplasmic	Periplasmic	Periplasmic	YES	NO	NO	Soluble
175.	ATH69300.1	Inner-Membrane	Cytoplasmic-Membrane	Inner-Membrane	NO	NO	Yes; 2 TM	Membrane; 2 TM
176.	ATH69301.1	Inner-Membrane	Cytoplasmic-Membrane	Inner-Membrane	NO	NO	Yes; 3TM	Membrane; 3 TM
177.	ATH69313.1	Outer membrane	Outer membrane	Outer membrane	YES	NO	Yes; 1TM	Membrane; 1 TM
178.	ATH69314.1	Periplasmic	Cytoplasmic-membrane	Periplasmic	YES	NO	Yes; 1TM	Membrane; 1 TM
179.	ATH69322.1	Cytoplasmic	Unknown	Cytoplasmic	NO	YES	NO	Soluble
180.	ATH69323.1	Inner-membrane	Cytoplasmic membrane	Inner-membrane	NO	NO	Yes; 3 TM	Membrane; 4 TM
181.	ATH69359.1	Periplasmic	Unknown	Inner-membrane	YES	NO	Yes; 1TM	Membrane; 1TM
182.	ATH69360.1	Inner-membrane	Cytoplasmic membrane	Inner-membrane	NO	NO	Yes; 4 TM	Membrane; 4 TM

Supplementary Table 3- Analysis of the subcellular localization and presence of transmembrane helices present in 246 HP's. Bioinformatic tools PSORTb, CELLO and PSLpred were used for subcellular localization; HMMTOP, SOSUI and TMHMM were used identifying soluble or membrane proteins with presence or absence of transmembrane helices; SignalP 4.1 and SecretomeP 2.0 were used for predicting signal peptide and secretory proteins.

183.	ATH69408.1	Periplasmic	Unknown	Periplasmic	YES	NO	Yes; 1TM	Membrane; 1TM
184.	ATH69432.1	Inner membrane	Cytoplasmic-Membrane	Inner membrane	NO	YES	Yes; 7TM	Membrane; 7 TM
185.	ATH69450.1	Cytoplasmic	Cytoplasmic	Cytoplasmic	NO	NO	NO	Soluble
186.	ATH69450.1	Periplasmic	Unknown	Periplasmic	YES	NO	NO	Soluble
187.	ATH69519.1	Periplasmic	Cytoplasmic-Membrane	Inner Membrane	NO	NO	Yes; 2TM	Membrane; 2 TM
188.	ATH69527.1	Periplasmic	Unknown	Periplasmic	YES	NO	NO	Soluble
189.	ATH69530.1	Outer Membrane	Unknown	Extracellular	YES	YES	Yes; 1TM	Membrane; 1TM
190.	ATH69548.1	Cytoplasmic	Cytoplasmic membrane	Inner-membrane	NO	NO	NO	Soluble
191.	ATH69552.1	Cytoplasmic	Unknown	Cytoplasmic	NO	NO	NO	Membrane; 1TM
192.	ATH69554.1	Inner-membrane	Cytoplasmic membrane	Inner-membrane	NO	NO	Yes; 3 TM	Membrane; 4 TM
193.	ATH69573.1	Periplasmic	Unknown	Inner-membrane	YES	NO	Yes; 1TM	Membrane; 1TM

Supplementary Table 3- Analysis of the subcellular localization and presence of transmembrane helices present in 246 HP's. Bioinformatic tools PSORTb, CELLO and PSLpred were used for subcellular localization; HMMTOP, SOSUI and TMHMM were used identifying soluble or membrane proteins with presence or absence of transmembrane helices; SignalP 4.1 and SecretomeP 2.0 were used for predicting signal peptide and secretory proteins.

194.	ATH69585.1	Cytoplasmic	Unknown	Cytoplasmic	NO	YES	NO	Soluble
195.	ATH69653.1	Cytoplasmic	Unknown	Inner-membrane	YES	NO	YES; 2TM	Membrane; 2 TM
196.	ATH69662.1	Extracellular	Unknown	Extracellular	NO	NO	NO	Soluble
197.	ATH69677.1	Periplasmic	Unknown	Periplasmic	YES	YES	NO	Membrane; 1TM
198.	ATH69683.1	Inner-membrane	Cytoplasmic membrane	Inner-membrane	NO	NO	Yes; 4 TM	Membrane; 4 TM
199.	ATH69717.1	Extracellular	Outer Membrane	Extracellular	YES	YES	Yes; 1TM	Membrane; 1TM
200.	ATH69741.1	Extracellular	Unknown	Cytoplasmic	NO	NO	NO	Soluble
201.	ATH69743.1	Cytoplasmic	Cytoplasmic	Cytoplasmic	NO	NO	NO	Soluble
202.	ATH69744.1	Cytoplasmic	Unknown	Cytoplasmic	NO	YES	NO	Soluble
203.	ATH69782.1	Periplasmic	Periplasmic	Periplasmic	YES	NO	NO	Soluble
204.	ATH70654.1	Inner-Membrane	Cytoplasmic-Membrane	Inner-Membrane	NO	NO	Yes; 2 TM	Membrane; 2 TM

Supplementary Table 3- Analysis of the subcellular localization and presence of transmembrane helices present in 246 HP's. Bioinformatic tools PSORTb, CELLO and PSLpred were used for subcellular localization; HMMTOP, SOSUI and TMHMM were used identifying soluble or membrane proteins with presence or absence of transmembrane helices; SignalP 4.1 and SecretomeP 2.0 were used for predicting signal peptide and secretory proteins.

205.	ATH69795.1	Periplasmic	Unknown	Periplasmic	YES	NO	YES 1TM/NO	Soluble
206.	ATH69815.1	Cytoplasmic/ Extracellular	Cytoplasmic membrane	Cytoplasmic	YES	NO	NO	Soluble
207.	ATH69824.1	Cytoplasmic	Cytoplasmic	Cytoplasmic	NO	NO	NO	Soluble
208.	ATH69836.1	Outer membrane	Extracellular	Periplasmic	NO	NO	NO	Soluble
209.	ATH69872.1	Extracellular	Unknown	Extracellular	NO	NO	NO	Soluble
210.	ATH70659.1	Cytoplasmic	Cytoplasmic	Cytoplasmic	NO	YES	NO	Soluble
211.	ATH70660.1	Periplasmic	Unknown	Periplasmic	YES	NO	YES 1TM/NO	Soluble
212.	ATH69906.1	Cytoplasmic/ Extracellular	Cytoplasmic membrane	Cytoplasmic	YES	NO	NO	Soluble
213.	ATH69913.1	Cytoplasmic	Cytoplasmic	Cytoplasmic	NO	NO	NO	Soluble
214.	ATH69954.1	Inner-Membrane	Cytoplasmic-Membrane	Inner-Membrane	NO	NO	Yes; 2 TM	Membrane; 2 TM
215.	ATH69997.1	Inner membrane/ Cytoplasmic	Cytoplasmic	Cytoplasmic	NO	YES	NO	Soluble

Supplementary Table 3- Analysis of the subcellular localization and presence of transmembrane helices present in 246 HP's. Bioinformatic tools PSORTb, CELLO and PSLpred were used for subcellular localization; HMMTOP, SOSUI and TMHMM were used identifying soluble or membrane proteins with presence or absence of transmembrane helices; SignalP 4.1 and SecretomeP 2.0 were used for predicting signal peptide and secretory proteins.

216.	ATH70006.1	Inner-Membrane	Cytoplasmic-Membrane	Inner-Membrane	NO	NO	Yes; 3TM	Membrane; 3 TM
217.	ATH70008.1	Inner-membrane	Cytoplasmic membrane	Inner-membrane	NO	NO	Yes 3 TM	Membrane; 3 TM
218.	ATH70042.1	Inner - Membrane	Cytoplasmic Membrane	Periplasmic Protein	YES	NO	NO	Soluble
219.	ATH70056.1	Extracellular	Unknown	Extracellular	NO	NO	NO	Soluble
220.	ATH70067.1	Inner-membrane	Cytoplasmic membrane	Inner-membrane	NO	NO	Yes; 10 TM	Membrane; 10 TM
221.	ATH70101.1	Periplasmic	Periplasmic	Periplasmic	NO	YES	Yes; 1 TM	Membrane; 1 TM
222.	ATH70117.1	Cytoplasmic	Unknown	Cytoplasmic	NO	NO	NO	Soluble
223.	ATH70671.1	Cytoplasmic	Cytoplasmic membrane	Inner-membrane	NO	NO	Yes; 1 TM	Membrane; 1 TM
224.	ATH70198.1	Cytoplasmic	Cytoplasmic	Cytoplasmic	NO	NO	NO	Soluble
225.	ATH70203.1	Outer Membrane	Outer Membrane	Outer Membrane	NO	YES	NO	Soluble
226.	ATH70213.	Cytoplasmic	Outer	Outer Membrane	NO	NO	NO	Soluble

Supplementary Table 3- Analysis of the subcellular localization and presence of transmembrane helices present in 246 HP's. Bioinformatic tools PSORTb, CELLO and PSLpred were used for subcellular localization; HMMTOP, SOSUI and TMHMM were used identifying soluble or membrane proteins with presence or absence of transmembrane helices; SignalP 4.1 and SecretomeP 2.0 were used for predicting signal peptide and secretory proteins.

	1		Membrane					
227.	ATH70218.1	Cytoplasmic	Cytoplasmic	Cytoplasmic	YES	NO	NO	Soluble
228.	ATH70219.1	Periplasmic	Unknown	Periplasmic	YES	NO	NO	Soluble
229.	ATH70237.1	Periplasmic	Unknown	Periplasmic	YES	NO	NO	Soluble
230.	ATH70244.1	Periplasmic	Cytoplasmic-Membrane	Inner Membrane	NO	NO	Yes; 2TM	Membrane; 2 TM
231.	ATH70274.1	Periplasmic	Unknown	Periplasmic	YES	NO	NO	Soluble
232.	ATH70275.1	Inner membrane	Cytoplasmic-Membrane	Inner membrane	NO	YES	Yes; 7TM	Membrane; 7 TM
233.	ATH70277.1	Cytoplasmic	Cytoplasmic	Cytoplasmic	NO	NO	NO	Soluble
234.	ATH70286.1	Cytoplasmic	Cytoplasmic	Cytoplasmic	NO	YES	NO	Soluble
235.	ATH70287.1	Periplasmic	Unknown	Periplasmic	YES	NO	YES 1TM/NO	Soluble
236.	ATH70326.1	Cytoplasmic/ Extracellular	Cytoplasmic membrane	Cytoplasmic	YES	NO	NO	Soluble
237.	ATH70347.	Cytoplasmic	Cytoplasmic	Cytoplasmic	NO	NO	NO	Soluble

Supplementary Table 3- Analysis of the subcellular localization and presence of transmembrane helices present in 246 HP's. Bioinformatic tools PSORTb, CELLO and PSLpred were used for subcellular localization; HMMTOP, SOSUI and TMHMM were used identifying soluble or membrane proteins with presence or absence of transmembrane helices; SignalP 4.1 and SecretomeP 2.0 were used for predicting signal peptide and secretory proteins.

	1							
238.	ATH70373.1	Periplasmic	Extracellular	Extracellular	NO	YES	Yes; 1 TM	Membrane; 1 TM
239.	ATH70395.1	Periplasmic	Outer-membrane	Cytoplasmic	YES	NO	Yes; 1 TM	Membrane; 1 TM
240.	ATH70424.1	Cytoplasmic	Cytoplasmic	Cytoplasmic	NO	NO	NO	Soluble
241.	ATH70426.1	Periplasmic	Unknown	Inner-membrane	NO	YES	NO	Soluble
242.	ATH70436.1	Periplasmic	Unknown	Cytoplasmic	YES	NO	NO	Soluble
243.	ATH70444.1	Cytoplasmic	Unknown	Inner-membrane	NO	YES	NO	Soluble
244.	ATH70449.1	Periplasmic	Unknown	Periplasmic	NO	YES	NO	Soluble
245.	ATH70469.1	Inner-membrane	Cytoplasmic membrane	Inner-membrane	NO	NO	Yes; 4 TM	Membrane; 5 TM
246.	ATH70687.1	Inner-membrane	Cytoplasmic membrane	Inner-membrane	NO	NO	Yes; 4 TM	Membrane; 5 TM
